# BNPower: a power calculation tool for data-driven network analysis for whole-brain connectome data

**DOI:** 10.1162/imag_a_00099

**Published:** 2024-02-28

**Authors:** Chuan Bi, Thomas Nichols, Hwiyoung Lee, Yifan Yang, Zhenyao Ye, Yezhi Pan, Elliot Hong, Peter Kochunov, Shuo Chen

**Affiliations:** Maryland Psychiatric Research Center, Department of Psychiatry, University of Maryland School of Medicine, Baltimore, MD, United States; Big Data Institute, Li Ka Shing Centre for Health Information and Discovery, Nuffield Department of Population Health, University of Oxford, Oxford, United Kingdom; Department of Mathematics, University of Maryland, College Park, College Park, MD, United States; Division of Biostatistics and Bioinformatics, Department of Epidemiology and Public Health, University of Maryland School of Medicine, Baltimore, MD, United States; Department of Psychiatry and Behavioral Science, University of Texas Health Science, Houston, TX, United States

**Keywords:** brain connectome, data-driven, network analysis, power analysis, simulation

## Abstract

Network analysis of whole-brain connectome data is widely employed to examine systematic changes in connections among brain areas caused by clinical and experimental conditions. In these analyses, the connectome data, represented as a matrix, are treated as outcomes, while the subject conditions serve as predictors. The objective of network analysis is to identify connectome subnetworks whose edges are associated with the predictors. Data-driven network analysis is a powerful approach that automatically organizes individual predictor-related connections (edges) into subnetworks, rather than relying on pre-specified subnetworks, thereby enabling network-level inference. However, power calculation for data-driven network analysis presents a challenge due to the data-driven nature of subnetwork identification, where nodes, edges, and model parameters cannot be pre-specified before the analysis. Additionally, data-driven network analysis involves multivariate edge variables and may entail multiple subnetworks, necessitating the correction for multiple testing (e.g., family-wise error rate (FWER) control). To address this issue, we developed BNPower, a user-friendly power calculation tool for data-driven network analysis. BNPower utilizes simulation analysis, taking into account the complexity of the data-driven network analysis model. We have implemented efficient computational strategies to facilitate data-driven network analysis, including subnetwork extraction and permutation tests for controlling FWER, while maintaining low computational costs. The toolkit, which includes a graphical user interface and source codes, is publicly available at the following GitHub repository: https://github.com/bichuan0419/brain_connectome_power_tool

## 1 Introduction

In the past two decades, there has been a growing interest in the study of the functional brain connectome. The functional brain connectome refers to a comprehensive collection of brain functional connections, where functional connectivities (FCs) are utilized to describe the synchronization of brain functions. Many computational and statistical methods have been proposed to analyze the functional brain connectome, including group independent component analysis (Calhoun et al., 2009), seed-to-voxel approaches (Liao et al., 2014), graph theoretical methods (Bullmore & Sporns, 2009), and network methods (Zalesky et al., 2010). Despite the promising findings in the functional brain connectome studies, researchers have raised concerns about the potential occurrence of underpowered studies, primarily due to small sample sizes and the multivariate nature of the functional brain connectome data. These limitations can lead to false positive findings and less reproducible results (Marek et al., 2022). Therefore, it is crucial to carefully plan study designs and conduct power analyses to ensure the robustness and reproducibility of study findings.

Power analysis provides guidance regarding the likelihood of successfully detecting an expected effect size with a given sample size (Cohen, 2013), making it highly desirable for clinical and neuroscience research. Recently, tailored power analysis tools have been developed for neuroimaging data (e.g., connectome power), which have made substantial contributions to the field. For example, Fmripower (Mumford, 2012) has been introduced to facilitate power calculations for two-stage fMRI models, specifically addressing ROI effects and group studies. Another notable tool, Neuropower (Durnez et al., 2016), leverages the brain volume of activated regions and the average effect size (ES) within those regions in fMRI images, enabling comprehensive power analysis. Additionally, PowerMap (Joyce & Hayasaka, 2012) serves as a versatile neuroimaging power analysis tool, capable of generating power and sample size estimates in the form of a 3D image. Traditional power analysis tools such as G*power (Faul et al., 2007), SAS, and R have also successfully implemented power analysis for neuroimaging data (Carter et al., 2016; G. Chen et al., 2014; Chepkoech et al., 2016). It is worth noting that in existing power analysis for neuroimaging studies, each edge representing the brain region association is treated independently. Consequently, achieving reproducible results in brain connectome studies may require large sample sizes, potentially in the thousands (Marek et al., 2022). Furthermore, a recent review article (Helwegen et al., 2023) emphasizes the importance of considering the “network organization” in determining power in connectomics, in addition to factors such as sample size, effect size, and significance regions. However, integrating network/graph characteristics with power analysis is inherently difficult, as it requires the implementation of sophisticated statistical and network analysis methods.

We first introduce two different subnetwork analysis methods that can effectively address the complexities of network analysis and power calculations. These methods offer distinct approaches for exploring brain connectivity patterns and their associations with clinical or experimental conditions.

- Method 1 (M1, pre-specified subnetwork analysis): In this approach, we pre-define resting-state brain networks based on existing knowledge from literature prior to the data analysis (Grieder et al., 2018; Lord et al., 2011; McCutcheon et al., 2019). For example, previous studies suggest that the default mode network (DMN) and salience network are associated with a range of clinical conditions (Broyd et al., 2009; Palaniyappan & Liddle, 2012). Then, the analysis associated with the clinical condition can just focus on these two networks (DMN and salience). In this instance, statistical analysis will be performed for all connections (edges) within these two networks for the study sample.
- Method 2 (M2, data-driven network analysis). In this approach, our goal is to identify subnetworks of brain connections (edges) that are specifically associated with the clinical or experimental condition, also known as the predictor-of-interest related subnetworks. These subnetworks exhibit organized structures, such as cliques or k-partite subnetworks, and their significance is evaluated through network-level statistical inference (S. Chen et al., 2015, 2023; Wu et al., 2022; Zalesky et al., 2010). By employing this methodology, we can effectively capture and analyze cohesive patterns of connectivity within the brain that are specifically linked to the condition under investigation.

**Fig. 1.**
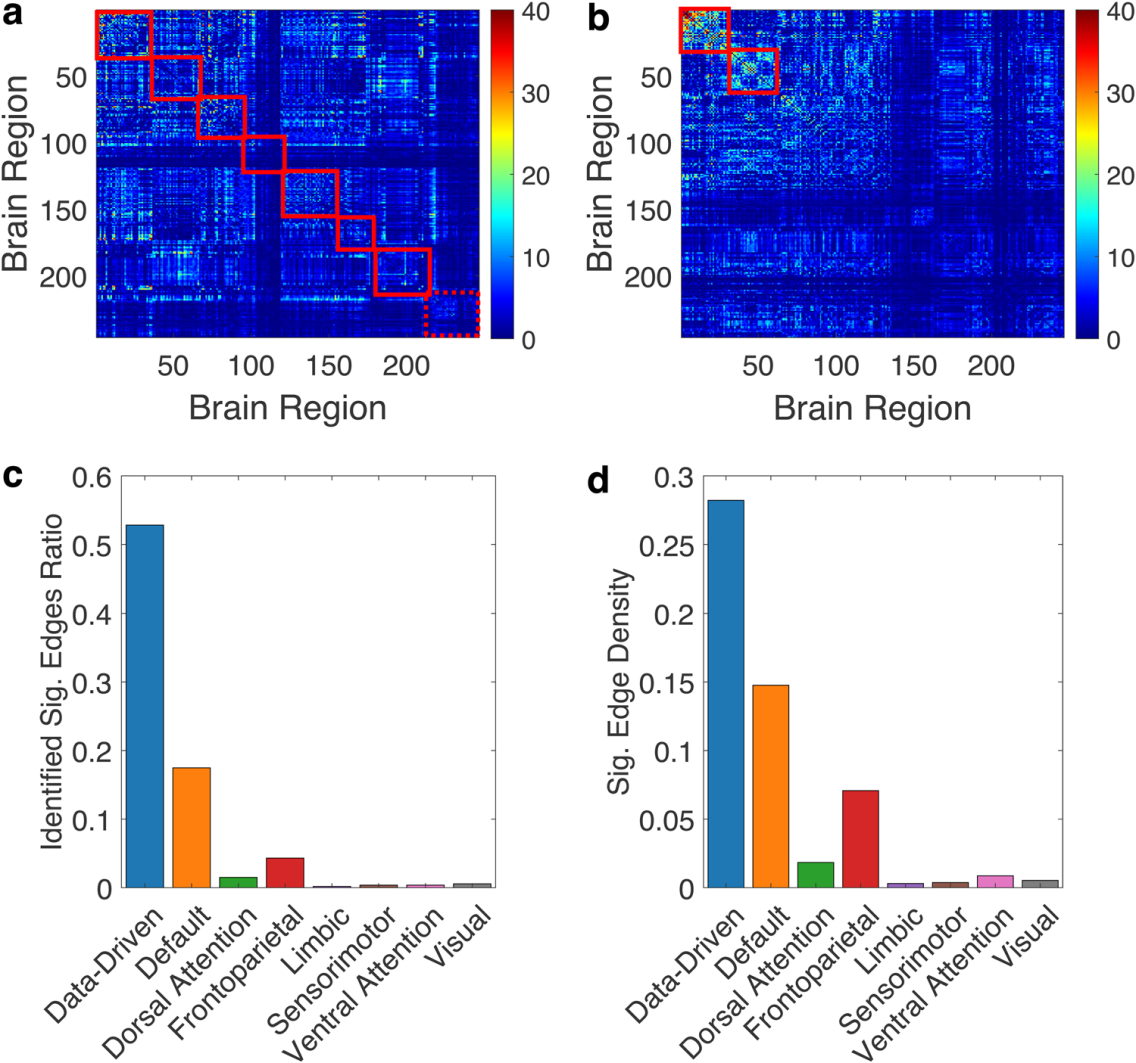
Distinguishable outcomes in brain connectome network analysis: a comparison of data-driven and pre-defined functional network approaches. The study employs a dataset from the UK Biobank, encompassing 40,926 subjects with usable pre-processed fMRI data. The investigated dependent variable is region-level functional connectivity (FC) (Fan et al., 2016), while the independent variable is the derived cognitive g-factor of the subjects (Mo et al., 2022). Two distinct analysis approaches, labeled as Approach M1 (a) and Approach M2 (b), are showcased. These approaches target cognitive g-factor-related FC subnetworks (submatrices), highlighted within red squares on the respective adjacency matrices. Each matrix element represents the −log ( p) value derived from association tests between each FC and the g-factor across subjects. In Approach M1, predefined networks based on Yeo’s 7-network parcellation Thomas (Yeo et al., 2011) were utilized. In contrast, Approach M2 employed a data-driven subnetwork detection method (Wu et al., 2022). Comparative summaries between the two approaches are shown in the bottom row. The x-axis represents subnetworks determined by Approaches M1 and M2. For Approach M1, subnetworks include default, dorsal attention, frontoparietal, limbic, sensorimotor, ventral attention, visual, and undefined networks. Approach M2 identifies two data-driven subnetworks. (c) illustrates the proportion of significant edges identified by Approaches M1 and M2, relative to the significant edges in the entire brain connectome. Significance is determined by weights surpassing a threshold based (S. Chen et al., 2023). (d) displays the ratio of significant edges within each subnetwork for Approach M1 and the two subnetworks extracted by Approach M2. We incorporated covariates such as age, sex, and head motion in our preliminary analysis. See Supplementary Material, Section 4 for more details.

M2 stands out as distinct from M1 due to its data-driven nature, as the predictor-of-interest-related subnetworks identified by M2 are derived directly from the data. On the other hand, in M1, subnetworks are predetermined prior to the analysis. While M1 allows for pre-specification of parameters and straightforward inference procedures, it may not fully capture the subnetworks associated with the predictor because the edges associated with the predictor may not fall within the pre-defined subnetworks. As a result, edges within the subnetworks identified by M1 are less likely to be associated with the predictor-of-interest, and conversely, edges associated with the predictor-of-interest may not be adequately covered by the subnetworks identified by M1. In contrast, M2 provides a more comprehensive characterization of the brain connectome changes associated with the predictor-of-interest (S. Chen et al., 2023; Wu et al., 2022). See Figure 1 for an example of the comparison between the two methods applied to real-life dataset.

### 1.1 Ethics statement

The demonstration showed in Figure 1 employed data sourced from the UK Biobank (UKB) project. Ethical clearance for the UKB was granted by the North West Multi-Centre Research Ethics Committee (MREC), documented under the approval number 11/NW/0382. Additionally, all individual participants involved in the UKB provided their written informed consent before participating in the study.

**Fig. 2.**
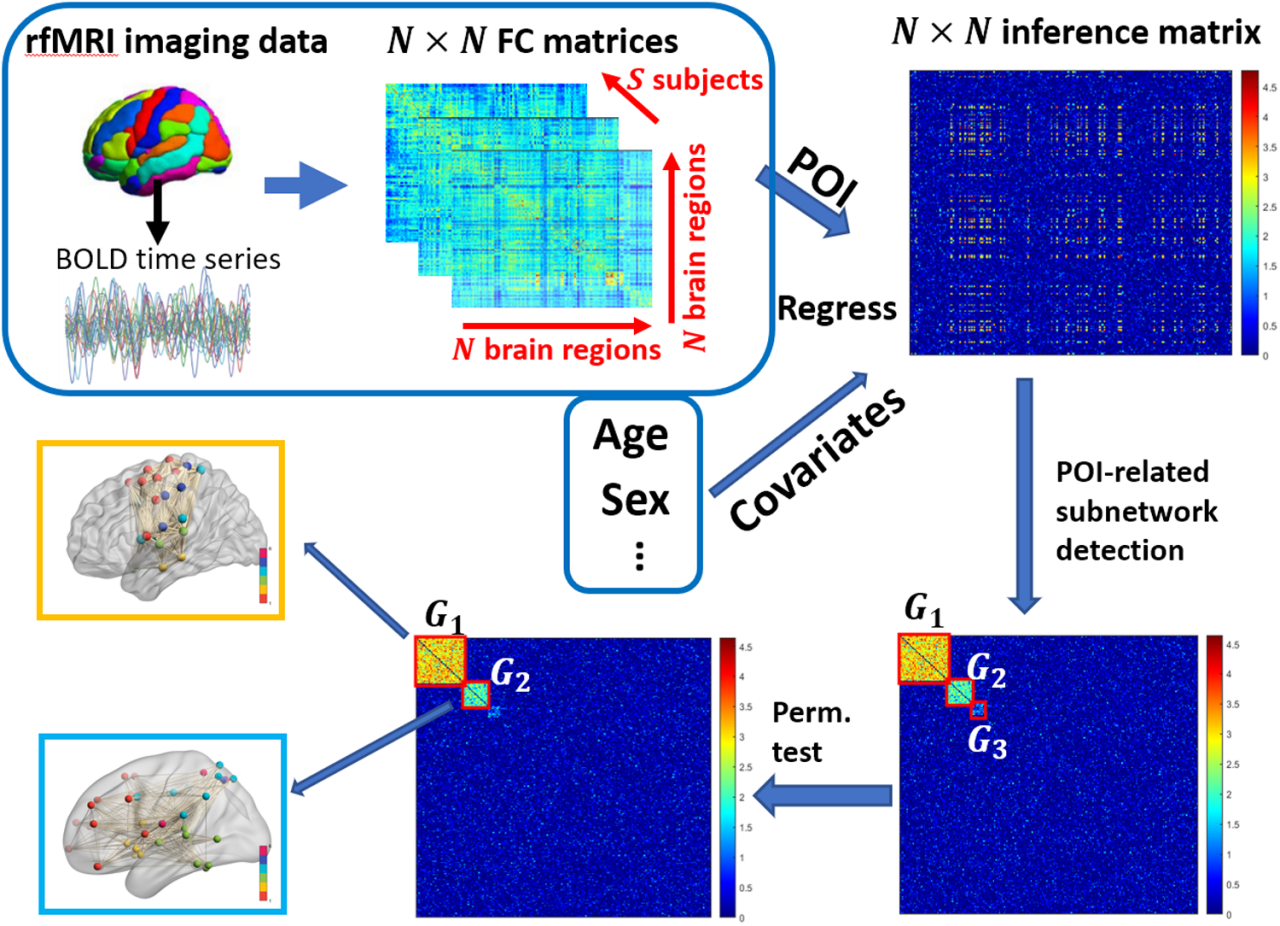
Pipeline for data-driven whole-brain network analysis. The pipeline involves calculating correlation-based functional connectivity (FC) matrices from region-level BOLD time series. Mass univariate regression analysis with covariates produces an inference matrix. A predictor-of-interest-related subnetwork detection algorithm identifies relevant subnetworks, followed by permutation tests for significance assessment. Visualization tools enable exploration of the detected subnetworks.

In this article, we use the term “data-driven network analysis” to refer to the network-level analysis of the functional brain connectome data using the data-driven approach, specifically M2. Data-driven network analysis involves the extraction and testing of subnetworks related to the predictor-of-interest from the entire brain connectome data. The data-driven network analysis procedure typically consists of three steps: i) Edge-wise inference: initially, we perform edge-wise inference to quantify the association between each connection (between pairs of brain areas) and the predictor-of-interest; ii) Subnetwork extraction: we extract organized subnetworks that exhibit a concentration of edges associated with the predictor-of-interest and possess a large number of nodes (S. Chen et al., 2015, 2023; Wu et al., 2022); iii) Subnetwork statistical testing: finally, we subject the predictor-of-interest-related subnetwork to statistical testing while controlling for FWER. The pipeline of data-driven network analysis for whole-brain connectome is illustrated in Figure 2. This data-driven approach allows us to identify edges specifically associated with the predictor-of-interest, resulting in the detection of organized subgraphs (e.g., cliques and k-partite graphs) that better reveal the systematic influence of the predictor-of-interest on the connectome. The primary focus of this study is to develop a power calculation method and the accompanying toolkit specifically designed for data-driven network analysis. The power calculation for data-driven network analysis is challenging. Firstly, unlike traditional power calculation approaches, the parameters for hypothesis testing in data-driven network analysis cannot be pre-specified prior to data analysis. This limitation makes commonly used power calculation software unsuitable for data-driven network analysis. Secondly, in data-driven network analysis, specifying predictor-of-interest-related subnetwork is essential for network-level inference and multiple testing correction. This requirement often involves permutation tests to account for the potential presence of multiple subnetworks. Lastly, the computational burden associated with data-driven network analysis can be substantial. The need for repeated simulations to estimate power can be time-consuming and computationally demanding.

To address these challenges, we developed a novel power calculation software of data-driven network analysis for whole-brain connectome data called Brain Network Power Calculator, or BNPower. BNPower utilizes a simulation-based approach that simulates the brain connectome data with latent predictor-of-interest-related subnetworks to estimate power. In contrast to classic simulation-based power analysis for complex models (e.g., generalized linear mixed models), BNPower takes into account graph characteristics such as subnetwork size and density in addition to the specification of effect sizes (e.g., Cohen’s d) on individual predictor-of-interest-related connections. BNPower implements data-driven network analysis on each simulated dataset, and calculating power as the proportion of successfully rejecting the null. We resort to computationally efficient strategies (e.g., greedy peeling algorithms) to circumvent the computational challenges, and further provide a friendly graphical user interface (GUI) for the general users.

## 2 Methods

### 2.1 Background: Power calculation for univariate neuroimaging outcome

First, we provide an introduction review of univariate power calculation. The statistical power is defined as the probability that a statistical hypothesis test correctly rejects the null hypothesis $H_{0}$ when the alternative hypothesis $H_{1}$ is true, which can be expressed mathematically as follows:

$$\text{power}=\mathbb{P}(\text{reject}H_{0}|H_{1}\text{is}\text{true})$$

Specifically, $H_{0}$ and $H_{1}$ are on basis of parameters in a statistical model, for example, testing a regression coefficient β=0. In the context of univariate imaging outcome inference, we have a generalized linear model that models the univariate outcome $Y^{s}$ for subject 1≤s≤S with P independent predictors $X,Z_{1},Z_{2},\cdots ,Z_{P-1}$, as shown in Equation (2.1):

$$g(E(Y^{s}))=\beta_{0}+\beta_{1}X^{s}+\beta_{2}Z_{1}^{s}+\cdots +\beta_{P}Z_{P-1}^{s},$$ (2.1)

where $\beta_{0}$ is the intercept, ${\left\{\beta_{i}\right\}}_{i=1}^{P}$ are weightings associated with variables ${\left\{X,Z\right\}}_{i=1}^{P-1},$ and *g* is the link function. We further denote *X^S^* to be the predictor-of-interest, and $Z_{1},Z_{2},\cdots ,Z_{P-1}$ are other related covariates such as demographic variables.

The power calculation requires the knowledge of three parameters: planned sample size, expected effect size, and the rejection region (e.g., α level). Consequently, the power can be determined by a closed formula in the case of a standard association analysis such as regression or t-test (see Supplementary Material, Section 1). For example, the power of two-sample t-test (a special case of (2.1)) is determined by the ES (Cohen’s d), SS of the two groups $S_{A}+S_{B}=S$, and α value (Harrison & Brady, 2004):

$$\begin{array}{l} \text{ power }=T_{S-2}\left(t_{\frac{\alpha }{2},S-2}|\frac{\text{Cohen’s }d}{\sqrt{\frac{1}{S_{A}}+\frac{1}{S_{B}}}}\right)\\ \;\;\;\;\;\;\;\;\;\;\;\;\;\;\;\;-T_{S-2}\left(-t_{\frac{\alpha }{2},S-2}|\frac{\text{Cohen’s }d}{\sqrt{\frac{1}{S_{A}}+\frac{1}{S_{B}}}}\right), \end{array}$$

where $t_{\alpha ,df}$ is the cut-off point determined from the central t-distribution given the level of significance α and degrees of freedom df, and $T_{df}\left(t_{\alpha ,df}|\delta \right)$ is the cumulative distribution function (CDF) of the non-central t-distribution $T_{df}\left(\cdot |\delta \right)$ associated with df and the non-centrality parameter δ, evaluated at $t_{\alpha ,df}$. Statistical computing software or packages are available for actual computations to guide the study design (Dupont & Plummer, 1997; Faul et al., 2007).

### 2.2 Data-driven whole-brain network analysis

In whole-brain network analysis, brain connectome data are often used as multivariate outcomes while clinical and experimental conditions serve as predictors. Let a weighted adjacency matrix $\text{Y}\in {\mathbb{R}}^{N\times N}$ denote brain connectome outcomes with $\frac{N\left(N-1\right)}{2}$ weighted edge variables, where N is the number of regions of interest (ROIs). An entry $Y_{ij}^{s}$ quantifies connection strength between the i-th and j-th brain regions (e.g., synchronization between two time series blood-oxygen-level-dependent, or BOLD signals for fMRI-based FC). We assume that ROIs are invariant across participants. Thus, a graph model G={V,E} is commonly used to characterize brain connectome topological structure, where the node set V represent ROIs (|V|=N) and the edge set E denote connections between ROIs. Like the regression model in (2.1), the predictor-of-interest X and covariates Z are independent variables. To assess the edge-wise association between FC outcomes and predictor-of-interest, the regression model is commonly used (S. Chen et al., 2023; Zhang et al., 2023).

In the study of functional brain connectome, the input data is a network comprising N nodes and $\frac{N\left(N-1\right)}{2}$ weighted edges, characterized by an N×N adjacency matrix denoted as $Y\in {\mathbb{R}}^{N\times N}$. For a subject s (s=1,⋯,S), we denote the connectome by a weighted graph $G^{s}=\left\{V,E,{\text{Y}}^{s}\right\}$, with |V|=N and $\left|E\right|=\frac{N\left(N-1\right)}{2}$, with edge weights in a weighted adjacency matrix $Y_{ij}^{s}$. Each element $Y_{ij}^{s}$ quantifies the synchronization (e.g., Pearson’s correlation coefficient) between two time series (blood-oxygen-level-dependent, or BOLD signals) of i and j-th brain region. The specific steps are:

*i.*  *Regression on individual edges.* The commonly used generalized matrix response regression model for whole-brain connectome outcome matrix ${\text{Y}}^{s}\in {\mathbb{R}}^{N\times N}$ reads (Zhang et al., 2023)
  $$g(E(Y_{ij}^{s}))=B_{0,ij}+B_{1,ij}X^{s}+B_{2,ij}Z_{1}^{s}+\cdots +B_{P,ij}Z_{P-1}^{s}$$ (2.2)
  where g is a link function and $X^{s}$ is the parameter of interest. Clearly, the statistical inference on $\left\{\beta_{ij}\right\}\;\;:=\left\{B_{1,ij}\right\}$ (e.g., mass univaraite test) does not automatically reveal the predictor-of-interest-related subnetwork, and a following network analysis procedure is required.
*ii.*  *Subnetwork extraction.* Upon the mass-univariate testing (e.g., $\beta_{ij}\neq 0$), we arrive at an N×N inference matrix W associated with $B_{1}$, where elements $w_{ij}$ represents association between the i-th and j-th brain regions. Without loss of generality, we use $-\text{log}\;\text{(}p_{ij}\text{)}$ values to represent the strength of the associations, where larger values correspond to stronger associations. Other values can also be considered such as test statistics, binarized values {0,1} based on a proper threshold. Moreover, matrix W is further characterized as the adjacency matrix associated with a weighted network G={V,E,W} that denotes the deferentially expressed whole-brain connectome that is associated with the predictor-of-interest. Let $G_{c}\subset G$ denote a subnetwork that consists of $\left|V_{c}\right|$ nodes and $\left|E_{c}\right|=\left(\frac{\left|V_{c}\right|}{2}\right)$ edges. $G_{c}$ is related to the predictor-of-interest if $\mathbb{P}(\beta_{ij}\neq 0|i,j\in G_{c})\gg \mathbb{P}(\beta_{ij}\neq 0|i,j\notin G_{c})$, which is generally an organized subgraph (e.g., clique or k-partite subgraph (S. Chen et al., 2020)). Since $G_{c}$ is unknown, we resort to dense subgraph extraction and network detection with $\ell_{0}$ shrinkage to estimate ${\hat{G}}_{c}$ from W (S. Chen et al. 2015, 2023; Wu et al., 2022).
*iii:*  *Statistical inference on extracted subnetworks.* Next, we perform statistical inference testing whether ${\hat{G}}_{c}$ is related to the predictor-of-interest. The null and alternative hypotheses are $H_{0}:{\hat{G}}_{c}$ is not related to the predictor-of-interest vs. $H_{1}:{\hat{G}}_{c}$ is related to the predictor-of-interest. However, the statistical inference for ${\hat{G}}_{c}$ is different from classic statistical inference because ${\hat{G}}_{c}$ are not pre-specified parameters such as $\beta_{ij}$. In our previous work, statistical inference methods have been established for ${\hat{G}}_{c}$ by leveraging graph combinatorics theories (S. Chen et al., 2023). In brief, the statistical significance of ${\hat{G}}_{c}$ is determined by both size and density of ${\hat{G}}_{c}$. The probability of rejecting the null is greater for a larger and denser subnetwork. Moreover, we control FWER for multiple ${\hat{G}}_{c}$ using the permutation test. We include the details of subnetwork extraction and statistical inference in the Supplementary Material, Section 2.

Unlike the power analysis for univariate that builds on statistical inference on clearly defined $\beta_{ij}\neq 0$, the power calculation of data-driven network analysis cannot be linked to pre-specified parameters because neither the nodes nor edges of ${\hat{G}}_{c}$ are known prior to the analysis. To address this issue, we adopt the commonly used simulation-based power analysis procedure for complex statistical models. In BNPower, the power analysis is based on edge-level (univariate) inference of two sample test and regression analysis corresponding to the two tabs in the BNPower GUI.

### 2.3 Simulation-based power analysis for data-driven network analysis

In this section, we will elaborate on the simulation-based procedure for the power calculation of data-driven network analysis. This procedure consists of three steps: i) simulate M brain connectome data sets under $H_{1}$; ii) perform statistical inference; and iii) calculate the power as the proportion of successfully rejecting the null hypothesis in ii) for all M datasets. The power analysis procedure is as follows.

Step 1. *Simulate FC and predictor-of-interest variables under the $H_{1}$*

1.1. Generate predictor-of-interest-related graph structure. Let $G=\cup_{c=1}^{C}G_{c}\cup G_{0}$ denote a general graph model, where each $G_{c}$ is a predictor-of-interest-related subnetwork such that $\mathbb{P}(\beta_{ij}\neq 0|i,j\in G_{c})\gg \mathbb{P}(\beta_{ij}\neq 0|i,j\notin G_{c})$ and the rest of G refers to $G_{0}$. First, we define the graph size of G by N nodes. Then, $\left|V_{c}\right|$ and $\left|V_{0}\right|$ are the sizes of subgraphs of $G_{c}$ and $G_{0}$ respectively, where $N=\sum_{c}^{C}\;\;\left|V_{c}\left|+\right|V_{0}\right|$. We next let $\rho_{1}=\mathbb{P}(\beta_{ij}\neq 0|i,j\in G_{c})$ and $\rho_{0}=\mathbb{P}(\beta_{ij}\neq 0|i,j\notin G_{c})$ with $\rho_{1}>\rho_{0}$. Using all above parameters, we determine edges with $\beta_{ij}\neq 0$ and $\beta_{ij}=0$. The required input parameters for this step are: N, $V_{c}$, $V_{0}$, $\rho_{1}$, and $\rho_{0}$.

1.2. Simulate FC matrices for S subjects with given sample size, effect size, and graph structure. We first specify the $\left\{X^{s}\right\}$ as the predictor-of-interest, where $X^{s}$ is categorical for group comparisons and continuous for regression analysis in (2.2). For all edges $\beta_{ij}\neq 0$, the connections are associated with the predictor-of-interest. On an edge with $\beta_{ij}\neq 0$, we let $E(Y_{ij}^{s}|X^{s})=b_{ij}+\beta_{ij}X^{s}$, where $\kappa_{ij}$ is the intercept (covariates can be further included as needed). The standardized ES of predictor-of-interest-related edges is jointly determined by $\beta_{ij}$ and variance parameters $\sigma_{ij}^{2}$. Without loss of generality, normal distribution is used for commonly used connectome metrics (Lee & Frangou, 2017). Then, we sample $Y_{ij}^{s}\sim N(E(Y_{ij}^{s}|X^{s}),\sigma_{ij}^{2})$ across all S participants, where for non-predictor-of-interest-related edges, we set the standard deviation of $Y_{ij}^{s}$ to σ. For two sample comparison, the Cohen’s d is simply $\frac{\beta_{ij}}{\sigma_{ij}}$. For continuous $X^{s}$, Cohen’s $f^{2}=\frac{\eta^{2}}{1-\eta^{2}}$, where η is the partial correlation coefficient between $X^{s}$ and $Y^{s}$. Repeating the above sampling procedure for all edges, we obtain $\left\{Y^{s},X^{s}\right\}$ for all S subjects.

Step 2. *Perform statistical inference*

2.1. Calculate inference matrix. Given the user-defined inputs (t-test or regression), the FC matrices $\left\{Y^{s}\right\}$ and predictor-of-interest $\left\{X^{s}\right\}$ for all subjects are determined. The FC matrices undergo a Fisher’s z-transform to ensure that the FC distributions exhibit normality. The mass-univariate testing (t-test or regression) will yield a weighted network G characterized by an inference matrix W of −log( p) values.

2.2. Extract ${\hat{G}}_{c}$. To identify ${\hat{G}}_{c}$, we use a greedy-peeling based algorithm (Charikar, 2003; S. Chen et al., 2023; Tsourakakis et al., 2013; Wu et al., 2022). For detailed implementation of the algorithm, see Supplementary Material, Section 2.1.

2.3. Conduct permutation test. Once ${\hat{G}}_{c}$ is obtained, we then shuffle group labels (or subject ID’s for regression), and repeat the above testing procedures 1.3 and 2.1 and generate test statistics for simulated data ${\left\{T\left({\hat{G}}_{c,m}^{R}\right)\right\}}_{m=1}^{M}$ and original data $T({\hat{G}}_{c})$. The p-value associated with ${\hat{G}}_{c}$ is the ranking of $T({\hat{G}}_{c})$ among ${\left\{T\left({\hat{G}}_{c,m}^{R}\right)\right\}}_{m=1}^{M}$. For details of the test statistics used, see Supplementary Material, Section 2.2.

2.4. Decide to accept or reject $H_{0}$ based on the α-level (e.g., 0.05).

Step 3. *Calculate statistical power*

Repeat the aforementioned steps 1 and 2 for K times. Therefore, the statistical power can be estimated as the ratio of the number of tests that correctly reject the $H_{0}$:

$$power=\frac{\text{Num}.\;\;\text{of tests to successfully reject }H_{0}}{K}$$ (2.3)

**Fig. 3.**
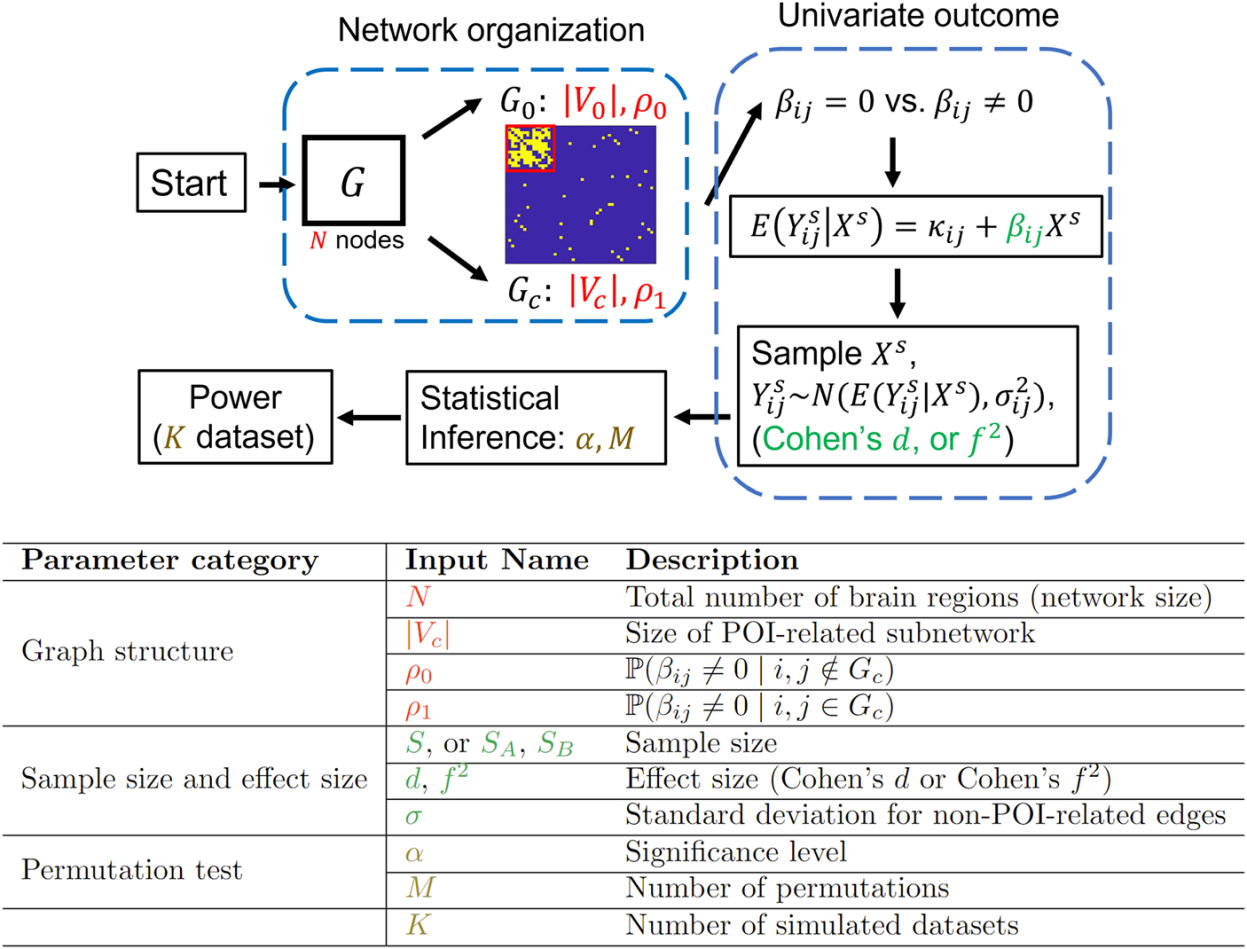
The pipeline for power calculation in BNPower. Top: a schematic depiction of the process for power calculation of the network outcome. Bottom: description of the input variables, for $\beta_{ij}=0$, we set the standard deviation for the non-predictor-of-interest-related edges to σ.

The complete process is graphically summarized in Figure 3.

As described above, the power calculation for the network outcome is determined by the SS S, level of significance α, and effect sizes (Cohen’s d or $f^{2}$), which are the same as univariate cases. Additionally, users need to specify the network-specific parameters, such as $N,\left|V_{c}\right|,\rho_{0},\rho_{1}$. We further allow the user to input the covariance matrix of FC variables which can be derived from existing FC datasets. In addition, we provided a dropdown menu to allow users to input the pre-defined reliability matrix (Helwegen et al., 2023) to more accurately assess the power (see Supplementary Material, Section 6 for derivations and demonstrative examples). In accordance with Helwegen et al. (2023), these required parameters are used to characterize the “network organization.” In addition, to better approximate the statistical power, the number of repetitions K and permutation tests M also helps determine the quality of obtained power. Since the null hypothesis states that ${\hat{G}}_{c}$ is not related to the predictor-of-interest, meaning no subnetwork is related to the predictor-of-interest, the power of identifying one network is essentially the same as identifying multiple subnetworks. Therefore, our power is calculated based on one predictor-of-interest-related subnetwork. In addition, given that there are various methods to assess the significance of subnetworks, such as Network-based Statistics (NBS), users can modify the code corresponding to step 2 in the aforementioned power calculation steps (See Supplementary Material, Section 6 for demonstration example). We summarize the description of input parameters for BNPower in Figure 3.

## 3 Results

### 3.1 Power calculation

The tool offers power calculation for two types of statistical tests at the network level—two-sample test and regression, which will be discussed separately along with worked examples.

#### 3.1.1 Two-sample test

**Fig. 4.**
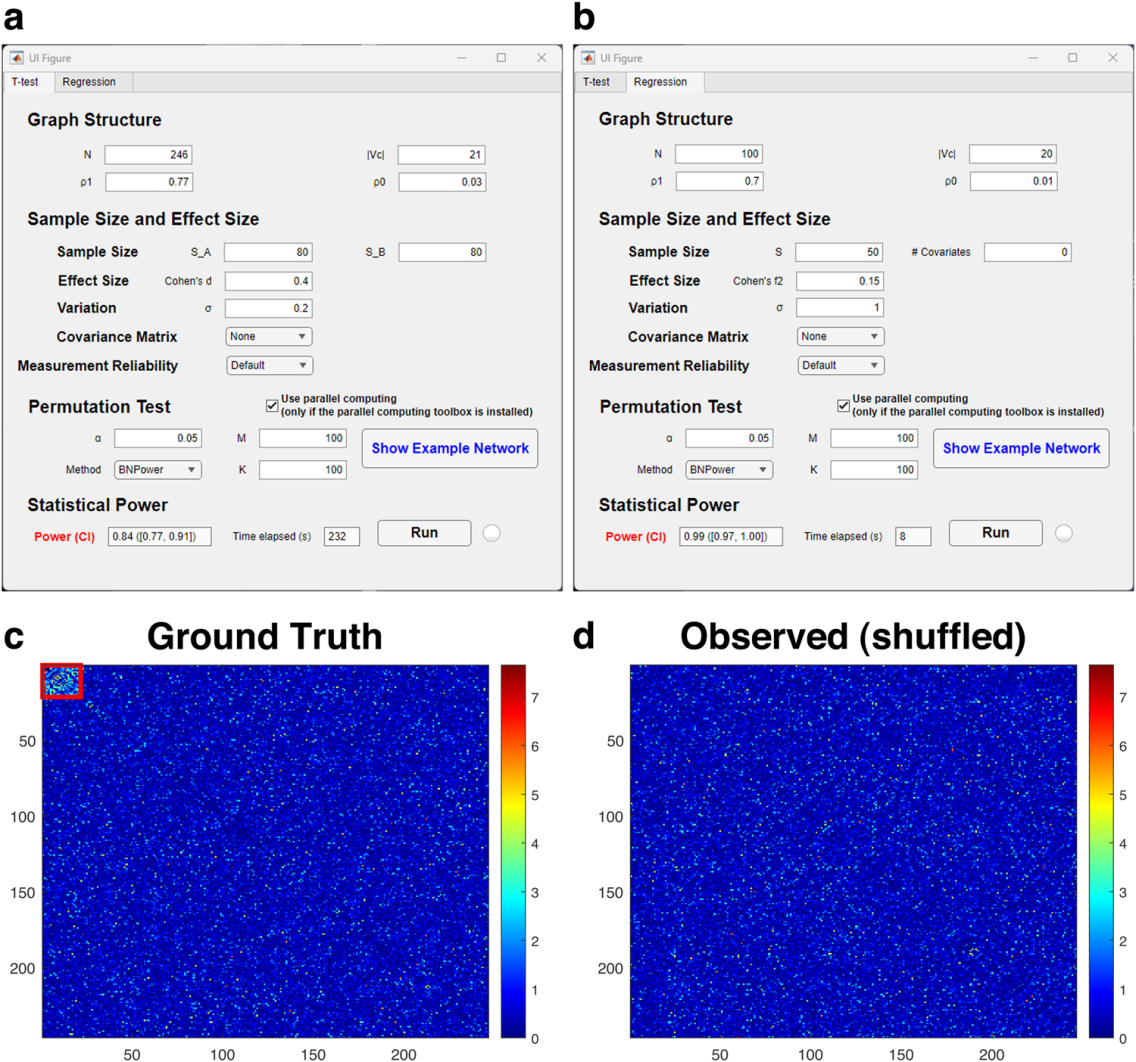
Graph user interface of BNPower. The upper section depicts the user interface of BNPower, showcasing its graphical layout (top), while providing illustrative instances of network adjacency matrices generated upon selecting the “Show Example Network” button (bottom). (a) illustrates the interface designed for power calculation in a two-sample test scenario, while (b) displays the corresponding interface tailored for power calculation in regression analysis. (c) showcases the ground truth, featuring a subnetwork linked to covariates, with emphasis on the top-left corner. In contrast, (d) presents an observed adjacency matrix with a shuffled node order. BNPower requires input across four parameter categories: the subnetwork’s graph structure pertinent to a predictor of interest is defined by N, $\left|V_{c}\right|$, $\rho_{0}$, and $\rho_{1}$; each functional connectivity (FC) entity, whether tied to the predictor of interest or not, is shaped by sample size, effect size, and variation. For each simulated dataset, M permutation tests are executed at a given significance level α, culminating in the statistical power figure displayed within the highlighted box after undergoing K repeated simulations.

The working GUI for two-sample test in BNPower is shown in Figure 4, which includes four categories of input parameters required from user to obtain the statistical power. The tool requires the user to first input parameters that are related to graph-structure of the predictor-of-interest-related subnetwork (see step 1.1 in Section 2.3), which in specific, the total number of brain regions (nodes) N, size of the predictor-of-interest-related subnetwork $\left|V_{c}\right|$ (with $\left|V_{c}\left|+\;\right|V_{0}\right|=N$ if the number of predictor-of-interest-related subnetwork is 1), the ratio of predictor-of-interest-related edges within and outside $G_{c}$, $\rho_{1}$ and $\rho_{0}$. After inputting the graph-structure related parameters, the differentially expressed brain connectome structure is determined.

In the example shown in Figure 4, we set N=246, $\left|V_{c}\right|=21$, $\rho_{1}=0.77$, and $\rho_{0}=0.03$. The second category of parameters are identical to what are needed for the univariate-outcome power calculation, that is, SS ($S_{A}$, $S_{B}$ for two clinical groups), standardized ES (Cohen’s d), and variation σ=0.2 in the derived FC. These parameters determine the FCs for predictor-of-interest-related edges, and for non-predictor-of-interest-related edges, the ES is 0. The aforementioned parameters are derived from the real-world dataset (UK biobank) on the study of identifying the aging-related FC subnetwork using the two-sample test (see Supplementary Material, Section 4 for details). After inputting the first two categories of parameters, the tool is ready to simulate FC matrices for each subject. In the worked example, we set $S_{A}=S_{B}=80$, Cohen’s d=0.4, σ=0.2.

After simulating the FC matrices, the mass-univariate two-sample test is performed on the simulated FC matrices that yield the inference matrix G. A “Show Example Network” button (highlighted in blue) is conveniently included for the user to inspect an example inference matrix before jumping into the statistical inference procedures; see Figure 4 for the previously input parameters. The predictor-of-interest-related subnetwork extraction algorithm is then performed to identify the predictor-of-interest-related subnetwork ${\hat{G}}_{c}$ (S. Chen et al., 2023; Charikar, 2003; Tsourakakis et al., 2013; Wu et al., 2022). Together with M (input by user) permutation tests, the decision on the null hypothesis is made given the user-input parameters α is made. By default, the number of permutation tests is set to be M=100.

Last, after specifying the number of repeated Monte-Carlo simulations K (e.g., 100 in the worked example), the statistical power will be calculated according to (2.3) and returned to user in the “Power” field (highlighted in red) as the output of the program. See Figure 4; as a result, the statistical power is approximately 0.84±0.07.

#### 3.1.2 Regression

The working GUI for regression in BNPower is shown in Figure 4. Same categories of parameters are required from the user as in the two-sample test. The power calculation for regression analysis requires the same parameters for graph-structure, statistical inference from user, as for the two-sample test. The only difference for the input parameters is the ES, where Cohen’s $f^{2}$ is used for regression. In addition, following the commonly used strategy for power analysis that accounts for covariates (Champely et al., 2017), BNPower (regression tab) allows users to input the number of covariates (#  Covariates). In the worked example shown in Figure 4, if we set N=150, $\left|V_{c}\right|=30$, $\rho_{1}=0.35$, $\rho_{0}=0.03$, S=100, #  Covariates=0, Cohen’s $f^{2}=0.15$, σ=1, M=100, and K=100, the resulted statistical power for the study design is 0.99 with 95% confidence interval being [0.97,1].

Once the parameter values are determined, the power calculation will start to execute as soon as the “Run” button is pushed with a progress bar. To expedite the computation process, parallel computation is allowed if the Parallel Computing Toolbox is installed. Additionally, a table detailing the expected runtime for various sample sizes, S, and the total number of nodes, N, is provided in Section 5 of the Supplementary Material. The required toolboxes and compatible MATLAB versions can be found in the GitHub repository.

### 3.2 Power curves, effect size, and sample size estimation

The statistical tool BNPower employs data-driven techniques to derive power estimates from provided input values, enabling the creation of power curves for the specific statistical test under investigation. In contrast to conventional power analysis tools, where researchers typically explore power curves by varying effect sizes and sample sizes, the unique context of brain connectome studies introduces the network organization as an additional determinant of statistical power.

**Fig. 5.**
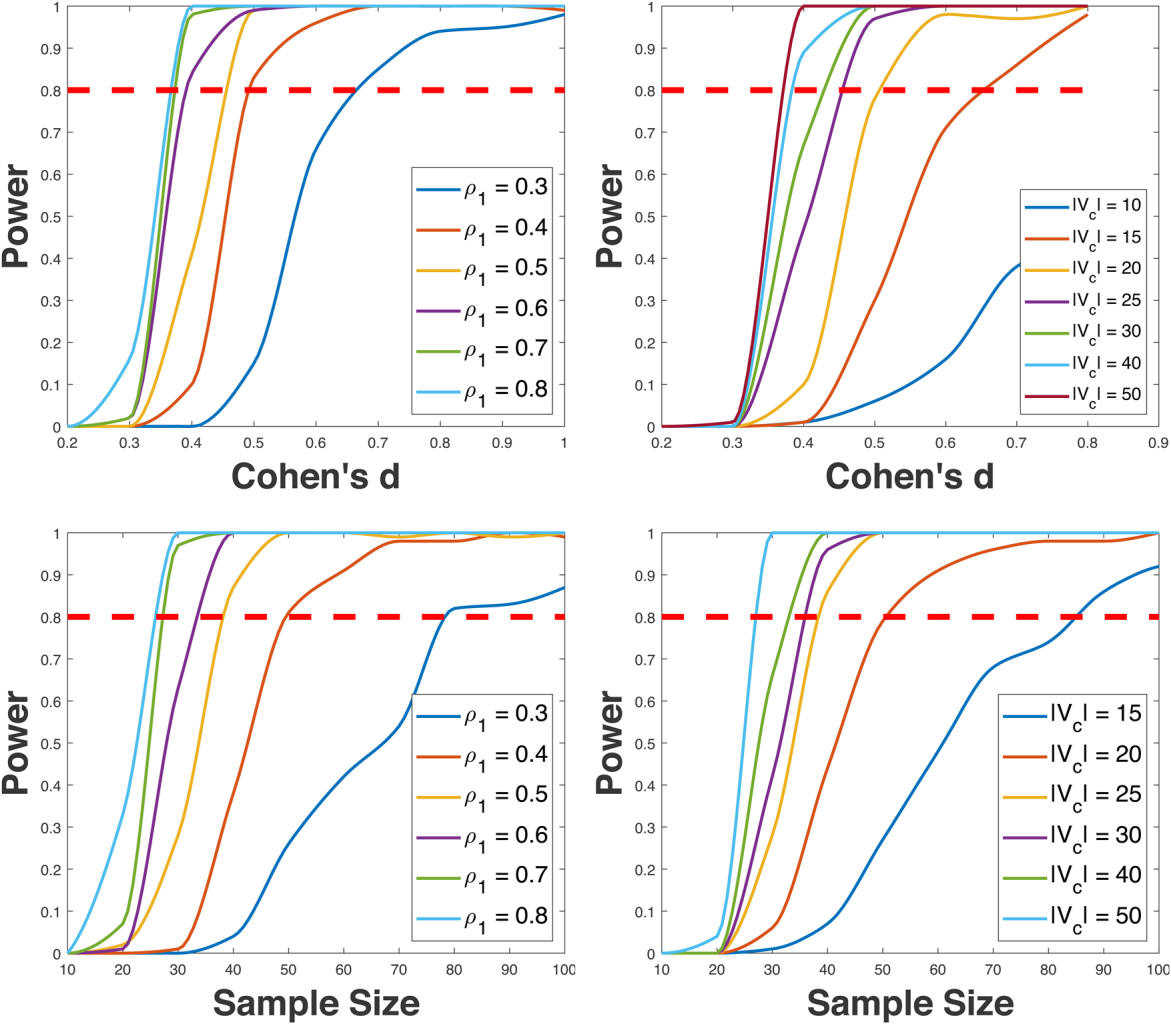
Power curves for two-sample test on network outcomes. The upper row of panels displays power curves illustrating the relationship between statistical power and effect size, while the lower row of panels showcases power curves depicting power as a function of sample size. The left and right panels present power curves obtained by varying $\rho_{1}$ and $\left|V_{c}\right|$ (the size of the predictor-of-interest subnetwork), respectively. The power curves are computed with default parameters: S=50 (sample size), N=100 (size of the entire network), $\left|V_{c}\right|=25$ (size of predictor-of-interest subnetwork), K=100 (number of repetitions per simulation), M=100 (number of permutation tests), Cohen’s d=0.5 (effect size), $\rho_{0}=0.02$, $\rho_{1}=0.5$, and α=0.05 (significance level).

To visualize the power curve generated by BNPower, we aggregate several power curves—such as power versus effect size/sample size—within a single panel. We systematically modify parameters (e.g., $\rho_{0}$, $\rho_{1}$, $\left|V_{c}\right|$) that impact network organization, allowing us to comprehensively assess their influence. Illustrative power curves are presented in Figures 5 for the two-sample test scenario. For regression analyses, corresponding power curves can be found in the Supplementary Material, specifically in Section 3.

The availability of these power curves facilitates the estimation of the minimum effect size (ES) or sample size (SS) needed to achieve a desired 80% power level while keeping other input parameters constant. Employing a grid search approach, we ascertain this minimum requirement. The process involves plotting the power curve associated with each candidate ES/SS value. The intersection point between the power curve and the horizontal line representing 80% power then indicates the minimum ES/SS. Refer to Figure 5 for a visual representation of this concept.

**Fig. 6.**
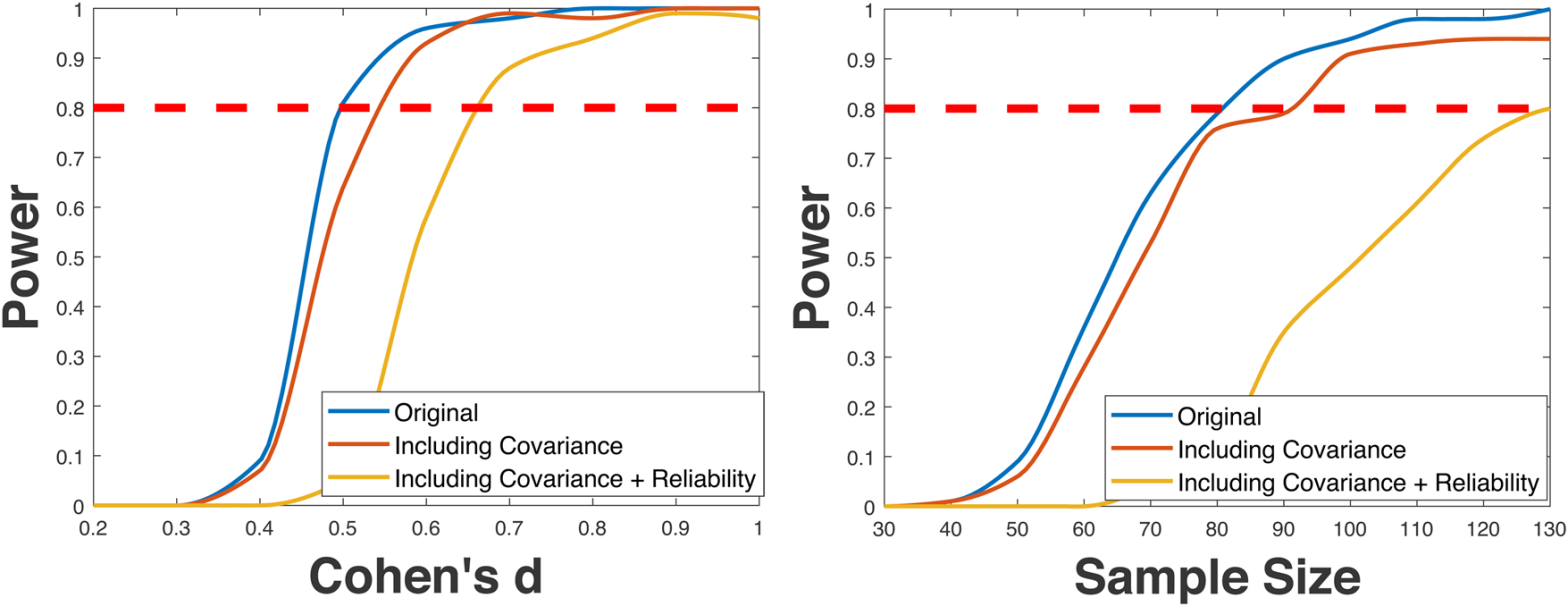
Evaluating the influence of covariance and reliability on the power analysis. We assess the effects on different effect and sample sizes. Generally, the power decreases by including covariance and reliability values in the simulation analysis.

Additionally, we demonstrate the influence of covariance and reliability values on power, as shown in Figure 6. We plot power curve comparisons between cases where no covariance or reliability values are included, only covariance is included, and both covariance and reliability values are included.

Generally, lower reliability values decrease the power because a higher intra-subject variability introduces additional variance (i.e., higher measurement errors) and thus reduces the efficiency of statistical inference. In addition, the simulation analysis that includes a covariance matrix can also potentially decrease the power because the covariance may disturb the accuracy of multivariate edge-level inference (see Fig. 6). Therefore, in practice, users may consider to increase the sample size to account for the factors of covariance and reliability.

## 4 Discussion

We have developed a toolkit named BNPower that performs statistical power analysis for human brain connectome data. The formal power analysis for brain connectome network data has been a challenge due to several key factors. Firstly, the inherent complexity of the brain connectome presents difficulties in establishing a specified structure and determining pre-specified parameters. Unlike traditional statistical analyses, where the variables and parameters are often explicitly defined, the intricate nature of brain connectivity necessitates a more flexible approach. Secondly, when examining the effect size in brain connectome analysis, it is not solely determined by a single parameter. Instead, factors such as subnetwork density and size also play crucial roles in shaping the observed effect. Consequently, capturing the true effect size becomes a multifaceted task, requiring a comprehensive understanding of the network’s characteristics and the interplay between various components. Lastly, the computation and control of FWER pose additional challenges in brain connectome power analysis. The sheer scale and complexity of brain connectome data demand graph $l_{0}$ shrinkage-based computational methods and techniques to ensure accurate and reliable results. Furthermore, the FWER, which involves accounting for permutation tests, becomes particularly intricate in this context, requiring careful consideration and advanced statistical approaches.

Our power analysis suggests different sample sizes in comparison to the sample sizes in BWAS (Marek et al., 2022). The difference is mainly driven by the different statistical inference methods. Unlike the mass univariate test in the BWAS paper (e.g., edge-wise corrected p<1e−7), the statistical inference data-driven network analysis is based on graph theory and combinatorics. The statistical theory suggests that the power of data-driven network analysis is determined by edge-level effect sizes, and the size and density of the predictor-related subnetwork. In other words, when predictor-related edges combine into a dense and relatively large (e.g., more than 10 nodes) subnetwork, a much smaller sample size is required for data-driven network analysis than mass univariate inference in BWAS. For example, we assume that the edge-level effect size is Cohen’s d=0.4. A sample size of 1000 is needed for BWAS with a threshold of $p<10^{-7}$. In contrast, only 160 participants are required for data-driven network analysis when predictor-of-interest related edges combined into a subnetwork with the sizes $\left|V_{c}\right|=21$, N=246, and densities $\rho_{1}=0.77$, $\rho_{0}=0.03$ (as shown in Section 3.1). This compelling evidence indicates that a smaller sample size than the traditionally accepted requirement of thousands of subjects is sufficient for achieving reliable and robust inference. The implications of our findings extend beyond the immediate scope of our power analysis. By demonstrating the feasibility of achieving reliable results with smaller sample sizes, we provide valuable guidance for future brain connectome analyses. Researchers can now consider more cost-effective and time-efficient study designs, as well as explore previously unattainable research questions due to the limitations imposed by large-scale data collection requirements. Moreover, our approach opens up new avenues for investigating specific data-driven subnetworks and their role in brain function and cognition. This fine-grained analysis at the subnetwork level not only enhances our understanding of the brain’s intricate workings but also paves the way for targeted interventions and personalized treatment strategies in fields such as neuroscience, psychiatry, and neurology.

Although we illustrate the application of BNPower to functional connectome network analysis, the tool is also applicable to other brain network analysis using EEG connectivity and white matter tractography connection data. In this study, we specifically employ functional connectivity as a demonstration tool to showcase the capabilities of our analysis tool. However, it is important to note that our tool is not limited to FC alone but is also applicable to a broader range of matrix response outcome analyses. For instance, our tool can seamlessly handle structural connectivity data, such as white matter probabilistic tractography. By leveraging the same principles and methodologies, we can explore the intricate connections and pathways within the brain’s white matter network. Furthermore, our tool extends its applicability to electroencephalography and magnetoencephalography connectome data acquired from multiple channels. Importantly, our method is not limited to a specific type of connectivity metric, including correlation-based metrics like Pearson correlation or more complex measures such as coherence, phase synchronization, or network efficiency. However, in a scenario where a few predictor-related edges span all nodes in a large, non-dense network, BNPower, set with subnetwork size equal to total node count and $\rho_{1}\approx \rho_{0}$, tends to yield low power (see Supplementary Material, Section 8).

Our method has the potential for further extensions and enhancements. For instance, it can be integrated with generalized linear models by incorporating appropriate links and distributional assumptions to accommodate non-normal or categorical outcome variables. This enables researchers to explore relationships between connectivity patterns and a wide range of response variables beyond traditional continuous measures. Moreover, our approach can be expanded to incorporate effect sizes, allowing researchers to quantify the strength and directionality of connectivity effects. This enhancement provides a deeper understanding of the impact of specific connections or subnetworks on the outcome of interest. Lastly, our method can also be extended to incorporate graph structure analysis, enabling researchers to explore network properties and topological characteristics within the connectome. This extension opens up avenues for investigating network centrality, modularity, small-worldness, or other graph-theoretical measures, providing additional insights into the organization and functional significance of brain connectivity patterns.

## Supplementary Material

Supplementary Material

## Data Availability

The stand-lone toolkit along with the source code and tutorial is freely available at https://github.com/bichuan0419/brain_connectome_power_tool.
