# Supplementary material for: BNPower: a power calculation tool for data-driven network analysis for whole-brain connectome data
Source: Imaging Neurosci (Camb). 2024 Feb 28;2:imag-2-00099. doi: 10.1162/imag_a_00099 (PMC12224405; doi:10.1162/imag_a_00099)
Supplement: Supplementary Material [file imag_a_00099-supp.pdf]

Supplementary material for "BNPower: a power calculation tool for data-driven network analysis for whole-brain connectome data"

Chuan Bi[1], Thomas Nichols[2], Hwiyoung Lee[1], Yifan Yang[5], Zhenyao Ye[1,3], Yezhi Pan[1], Elliot Hong[4], Peter Kochunov[4], and Shuo Chen*[1,3]

[1]Maryland Psychiatric Research Center, Department of Psychiatry, University of Maryland School of Medicine, Baltimore, MD, USA

[2]Big Data Institute, Li Ka Shing Centre for Health Information and Discovery, Nuffield Department of Population Health, University of Oxford, Oxford, UK

[3]Division of Biostatistics and Bioinformatics, Department of Epidemiology and Public Health, University of Maryland School of Medicine, Baltimore, MD, USA

[4]Department of Psychiatry and Behavioral Science, University of Texas Health Science, Houston, TX, USA

[5]Department of Mathematics, University of Maryland, College Park, College Park, MD, USA

February 3, 2024

*Corresponding author: ShuoChen@som.umaryland.edu

1 Review of statistical power analysis for univariate outcome

We first provide a brief review for the commonly used power analysis strategies for the two-sample test and regression of univariate-outcomes. We consider a general setting for univariate outcome inference, let $Y^s \in \mathbb{R}^1$ be the outcome variable for subject $1 \leq s \leq S$ in a sample of S participants. We denote X^s as the predictor of interest and $Z_1, \cdots, Z_{P-1}$ for the covariates.

For both the two-sample t-test and multiple linear regression, the power calculation is determined by the sample size S, level of significance α, and (standardized) effect sizes. For example, in the case of the two-sample t-test, we assume that X^s in Eq. (??) in the main document denotes two clinical groups A and B with sample sizes S_A and S_B, respectively. We further assume that the two populations are normally distributed, i.e., $Y_A^s \sim \mathcal{N}(\mu_0, \sigma_\epsilon)$ and $Y_B^s \sim \mathcal{N}(\mu_1, \sigma_\epsilon)$. The null hypothesis is $H_0 : \mu_1 - \mu_0 = 0$ and the alternative hypothesis is $H_1 : \mu_1 - \mu_0 \neq 0$. The resulting power can be obtained by Harrison and Brady (2004):

$$\text{power} = T_{S-2}\left(t_{\frac{\alpha}{2},S-2}\middle|\frac{\mu_1-\mu_0}{\sigma_\epsilon\sqrt{\frac{1}{S_A}+\frac{1}{S_B}}}\right) - T_{S-2}\left(-t_{\frac{\alpha}{2},S-2}\middle|\frac{\mu_1-\mu_0}{\sigma_\epsilon\sqrt{\frac{1}{S_A}+\frac{1}{S_B}}}\right), \tag{1.1}$$

where $t_{\alpha,df}$ is the cut-off point determined from the central t-distribution given the level of significance α and degrees of freedom df, and $T_{df}\left(t_{\alpha,df}|\delta\right)$ is the cumulative distribution function (CDF) of the non-central t-distribution $T_{df}\left(\cdot|\delta\right)$ associated with df and the non-centrality parameter δ, evaluated at $t_{\alpha,df}$. The non-centrality parameter δ can be obtained from the effect size (ES), e.g., Cohen's d for the t-test. For more complex models (e.g., generalized additive model), no closed-form power calculation formula is available and thus simulation-based power analysis is commonly used.

For multiple linear regression, the appropriate effect size to consider is Cohen's $f^2 = \frac{\eta^2}{1-\eta^2}$ where η is the partial correlation between X^s and the neuroimaging outcome. To calculate the statistical power for detecting any predictor that has at least one regression coefficient β_i significantly different from 0, we use a non-central F distribution, defined as follows:

$$\text{power} = 1 - F_{P,S-P-1}\left(f_{\alpha,P,S-P-1}\middle|f^2 \cdot S\right), \tag{1.2}$$

Here, $F_{df_1,df_2}\left(f_{\alpha,df_1,df_2}\middle|\delta\right)$ represents the non-central F cumulative distribution function with non-centrality parameter δ, evaluated at f_{α,df_1,df_2}. The critical value f_{α,df_1,df_2} is obtained from the central F-distribution function, which corresponds to df_1 numerator and df_2 denominator degrees of freedom such that $\mathbb{P}\left(F \leq f_{\alpha,df_1,df_2}\right) = \alpha$. The statistical power calculation is based on the sample size S, level of significance α, and effect size δ determined by the coefficient of determination η^2 in the regression model.

2 Statistical inference for predictor-of-interest-related subnetwork detection

2.1 Predictor-of-interest-related subnetwork detection algorithm

A previous study (Lemma 1 in Chen et al. Chen et al. (2023)) has demonstrated that under the null hypothesis, where no predictor-of-interest-related subnetwork exists, the probability of there being a large, dense subnetwork is almost 0. On the other hand, the probability of there being a large and dense subnetwork is almost 1 under the alternative hypothesis. Therefore, a subnetwork detection algorithm should aim to maximize a quantity that involves both the size and density of the subnetwork to be extracted, and should return a membership for each node Chen et al. (2023); Wu et al. (2022).

$$\{G_c\}_{c=1}^{C} = \underset{C^*}{\operatorname{argmax}} \left(\frac{\sum_{c=1}^{C^*}\sum_{i<j}(w_{i,j}|e_{i,j}\in G_c)}{\sum_{c=1}^{C^*}|E_c|}\right)^{\lambda}\left(\sum_{c=1}^{C^*}\sum_{i<j}(w_{i,j}|e_{i,j}\in G_c)\right)^{1-\lambda}, \tag{2.1}$$

Where C^* is the number of detected predictor-of-interest-related subnetworks, and λ is a tuning parameter that can be calculated through a grid search Chen et al. (2023). In other words, by maximizing the objective function in Equation (2.1), we can identify a subnetwork that contains the most predictor-of-interest-edges while controlling for false positives. For the sake of efficiency, we conveniently assume $\lambda = 0.5$ for the actual computation and adopt a greedy-peeling-based algorithm to maximize the objective function (2.1) Wu et al. (2022). It can be shown that the objective function is reduced to determining the predictor-of-interest-related subnetwork that maximizes the average degree. We are considering releasing a comprehensive version of BNPower that automatically optimizes λ, which will require a longer computation time.

2.2 Test statistics for predictor-of-interest-related subnetworks

Once the predictor-of-interest-related subnetworks $\hat{G}_c$ have been identified, we adopt a permutation-based test to approximate the distribution of test statistics associated with the subnetwork. For example, the corresponding null hypothesis H_0 and alternative hypothesis H_1 can be:

$$\begin{aligned} &H_0 : G_c \text{ is not a subnetwork related to } X, \\ &H_1 : G_c \text{ is related to } X. \end{aligned} \tag{2.2}$$

Under the null hypothesis, G_c is composed of false positive findings and, thus, is theoretically unlikely to be large and dense. Therefore, for a data sample, we perform permutation-based inference on each $\hat{G}_c$ to assess the statistical significance while controlling the family-wise error rate. The general procedure is similar to the permutation test in cluster-extent analysis Woo et al. (2014). However, unlike cluster-extent analysis, the testing object in our subnetwork inference is a subgraph rather than a subset of spatially connected suprathreshold voxels. To ensure the accuracy of the inference and account for graph combinatorics, we employ a network-level test statistic Chen et al. (2023):

$$T(\hat{G}_c) = \int 2N \cdot \exp\left\{ |\hat{G}_c|^2 \left\{ \frac{2}{(\hat{\gamma} - \hat{p})^2} + \frac{2}{3(\hat{\gamma} - \hat{p})} \right\}^{-1} \right\} g(r) dr. \tag{2.3}$$

In the expression (2.3) for the test statistics of the predictor-of-interest-related subnetwork, $g(r)$ represents a prior distribution of the cut-off values. Meanwhile, $\hat{p}$ and $\hat{\gamma}$ denote the edge densities for the overall network and the predictor-of-interest-related subnetwork, respectively, after binarization with an appropriate cut-off $\hat{r}$:

$$\hat{p} = \frac{\sum_{1 \le i < j \le N} I(w_{ij} > \hat{r})}{\binom{N}{2}}, \quad \hat{\gamma} = \frac{\sum_{(i,j) \in \hat{G}_c} I(w_{ij} > \hat{r})}{\binom{|\hat{V}_c|}{2}},$$

where the cut-off $\hat{r}$ is obtained in the data simulation.

Our network level statistic is jointly determined by the *size* and *density* of each extracted $\hat{G}_c$, which can better represent the combinatorial probability (rareness) of the observed network than network size alone Chen et al. (2023).

Lastly, we approximate the distributions of T under H_0 by performing K permutation tests Zalesky et al. (2010), and ascribe a p-value to G_c based on the ranking of $T(\hat{G}_c)$ among $T(\hat{G}_c^R)$.

3 Power curves for regression

Furthermore, we present power analysis curves for subnetwork analysis based on multiple linear regression. Differing from the conventional approach where researchers typically explore power curves by manipulating effect sizes and sample sizes, the intricacies of brain connectome studies introduce the pivotal influence of network organization on statistical power. To visually capture the power curve using BNPower, we aggregate various power curves (such as power versus effect size/sample size) within a unified panel, simultaneously adjusting parameters (e.g., ρ_0, ρ_1, $|V_c|$) that impact network organization. This allows a comprehensive assessment of the interplay between these parameters and power. The power curves specifically tailored for regression analysis are depicted in Figure 1.

4 Real-world examples from UK Biobank

4.1 Material and methods

Study samples

Our study involved the analysis of a cohort from the general population. The cohort was derived from the UK Biobank Sudlow et al. (2015) (UKB, `http://www.ukbiobank.ac.uk/`), an extensive prospective study encompassing around 500,000 individuals aged between 40 and 80 years, recruited from 2006 to 2010 at 22 different centers throughout the UK. The UKB study received ethical clearance from the National Information Governance Board for Health and Social Care as well as the NHS North West Multicenter Research Ethics Committee (REC reference 21/NW/0157). All participants in the UKB provided written consent. Around 100,000 of these participants were subjected to brain MRI examinations Alfaro-Almagro et al. (2018); Miller et al. (2016). Our study made use of the resting-state functional MRI (rfMRI) data, specifically from the v1.8 December 2020 dataset, which included approximately 43,000 individuals, starting from 2014. In our focus on the normal aging process, we excluded participants from the UKB cohort who had diagnoses of dementia, brain diseases, brain injuries, and mental disorders (totalling 1,248 individuals) as identified by ICD-10 codes. This resulted in a final sample size of approximately 41,000 subjects. Data for this study were obtained and research conducted under the UKB application license number 74376.

rfMRI acquisition, preprocessing and quality control

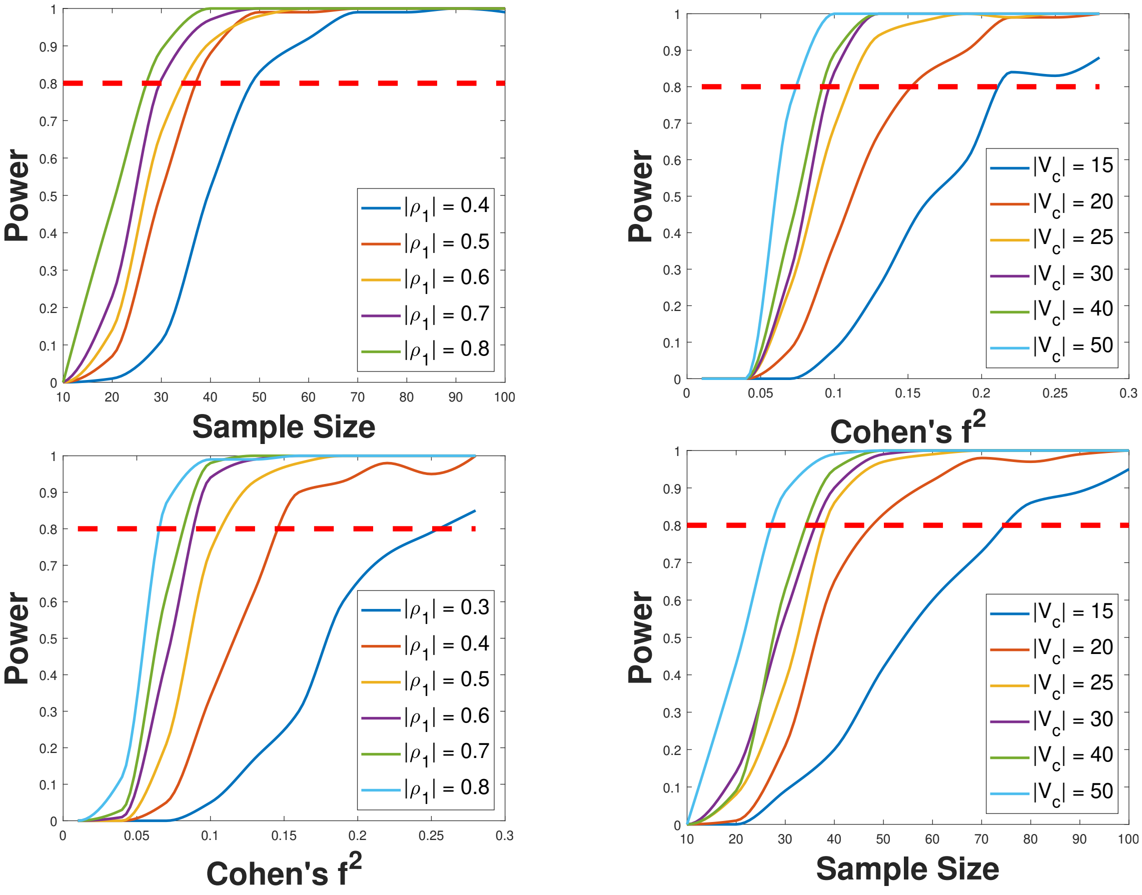

Figure 1: Power curves for Two-sample test on network outcomes. The upper row of panels displays power curves illustrating the relationship between statistical power and effect size, while the lower row of panels showcases power curves depicting power as a function of sample size. The left and right panels present power curves obtained by varying ρ_1 and $|V_c|$ (the size of the predictor-of-interest subnetwork), respectively. The power curves are computed with default parameters: $S = 50$ (sample size), $N = 100$ (size of the entire network), $V_c = 25$ (size of predictor-of-interest subnetwork), $\#\ Covariates = 0$, $K = 100$ (number of repetitions per simulation), $M = 100$ (number of permutation tests), Cohen's $f2 = 0.15$ (effect size), $\rho_0 = 0.02$, $\rho_1 = 0.5$, and $\alpha = 0.05$ (significance level).

In the UKB, MRI imaging was conducted using a Siemens Skyra 3-Tesla machine, equipped with a 32-channel RF receive head coil. The detailed protocols for these scans are accessible at https://biobank.ctsu.ox.ac.uk/crystal/crystal/docs/brain_mri.pdf. The rfMRI data collection spanned a 6-minute period (490 timepoints), characterized by a repetition time of 735ms, an echo time of 39ms, and a multi-band acceleration factor of 8. Each scan offered a 2.4mm isotropic resolution across 64 slices.

The rfMRI data obtained from the UKB were processed through several stages, starting with echo-planar imaging (EPI) unwarping and gradient distortion correction (GDC), followed by motion correction using MC-FLIRT Jenkinson et al. (2002). This step was crucial for minimizing interpolation artifacts. Post-correction, the data underwent a FIX cleaning process Salimi-Khorshidi et al. (2014), aimed at eliminating structural artifacts. Further processing involved aligning the rfMRI data to a 2mm MNI152-template Grabner et al. (2006) using the FSL software (FMRIB Software Library) Jenkinson et al. (2012). This alignment served the dual purpose of standardizing the brain images and filtering out non-cerebral regions while preserving areas of interest. Time-series data extraction for each of the 246 ROIs, based on The Human Brainnetome Atlas Fan et al. (2016b), was performed using the AFNI software (Analysis of Functional Neurolmages) Cox and Hyde (1997).

Quality assurance for the UKB imaging data adhered to a comprehensive, automated protocol Alfaro-Almagro et al. (2018). This protocol included the exclusion of subjects whose T1-weighted structural scans exhibited issues such as incomplete mapping to the standard space, excessive head motion, poor field or contrast quality, atypical structures, among others. A detailed description of the quality control criteria is documented in Alfaro-Almagro et al. (2018).

4.2 Demostration of pre-defined network analysis (M1) and data-driven network analysis (M2)

In BNPower, we implemented data-driven network analysis, as is demonstrated by the original results in Figure 1, data-driven network analysis outperforms pre-defined network analysis method in identifying the predictor-of-interest.

Dataset and Subjects

The study utilized a dataset from the UK Biobank, which included 40,926 subjects. These subjects had usable pre-processed functional Magnetic Resonance Imaging (fMRI) data.

Variables

The primary dependent variable in this study was region-level functional connectivity (FC), as described by Fan et al. (2016b). The independent variable was the cognitive g-factor derived from the subjects, following the methodology outlined by Mo et al. (2021).

Analytical Approaches

Two distinct analysis approaches were employed to investigate the relationship between FC and the cognitive g-factor:

- M1: This approach utilized predefined networks based on Yeo's 7-network parcellation Thomas Yeo et al. (2011). These networks included default, dorsal attention, frontoparietal, limbic, sensorimotor, ventral attention, visual, and undefined networks.

- M2: This approach employed a data-driven subnetwork detection method Wu et al. (2022). It focused on identifying predictor-of-interest related subnetworks.

Both approaches aimed to identify cognitive g-factor-related FC subnetworks within the brain's connectome network.

Statistical Analysis

Each matrix element in the study represented the negative logarithm of the p-value (-log(p)) derived from association tests between each FC and the g-factor across subjects. The significance of the connections was determined based on Chen et al. (2023).

Results

The study presented a comparative analysis of the two approaches in identifying significant edges within the brain's connectome related to the cognitive g-factor shown in Figure 1 in the main manuscript:

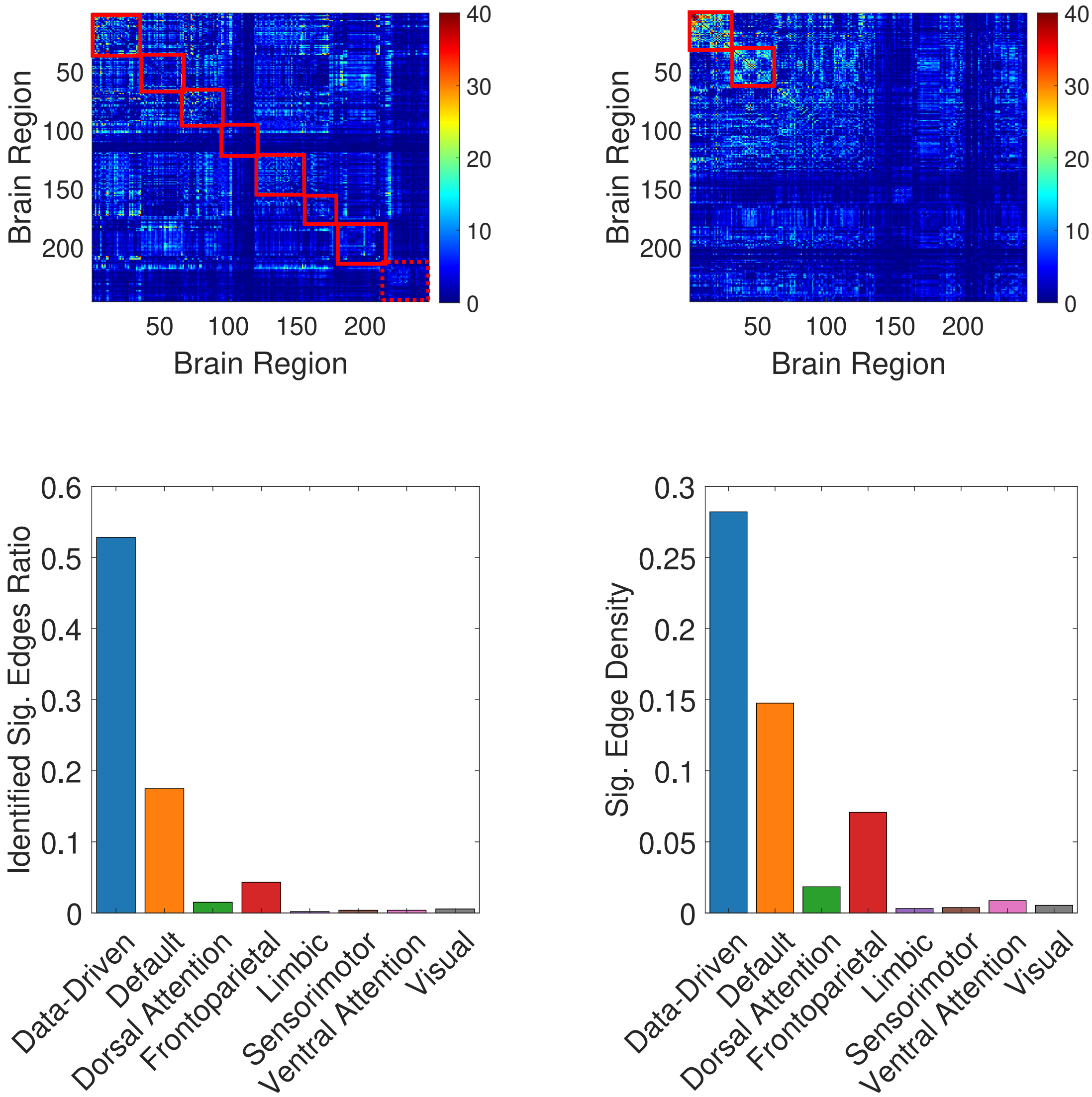

Figure 2: Distinguishable outcomes in brain connectome network analysis: a comparison of data-driven and pre-defined functional network approaches. Same as Figure 1 in the main manuscript.

- Proportion of Significant Edges: The bottom-left figure in the study illustrated the proportion of significant edges identified by both approaches relative to the significant edges in the entire brain connectome. This comparison highlighted the effectiveness of each approach in detecting relevant subnetworks.

- Subnetwork Analysis:

  – Bottom left figure (Significant Edge Ratio): It shows the ratio of edges (connections between brain regions) that are considered significant. The blue bar could represent the

results from Approach M2 and the rest from Approach M1, indicating that M2 found a higher proportion of significant connections.

– Bottom left figure (Significant Edge Density): This is showing the density of significant edges within the detected subnetworks. It follows the same color coding, with M2 showing a higher density compared to M1.

The results demonstrates the superiority of the data-driven approach in identifying predictor-of-interest related subnetworks. Approach M2, which uses data-driven networks, identifies more significant connections and denser networks than Approach M1, which is pre-defined. As a result, BNPower utilizes the data-driven network analysis approach to obtain simulation-based power at the network level.

4.3 Sample size calculation comparison using derived parameters for Aging-related subnetworks

The subjects used for the two-sample test for older and younger age groups are defined as follows. The older subject have age larger or equal to 75 years old, and the younger subjects are defined as 50 years old and younger. As a result, there are 2323 subjects in the older adults and 819 subjects in the younger group.

Parameter derivation

Using mass univariate two-sample test, we arrived at the inference map $(-\log_{10}(p))$ matrix as follows:

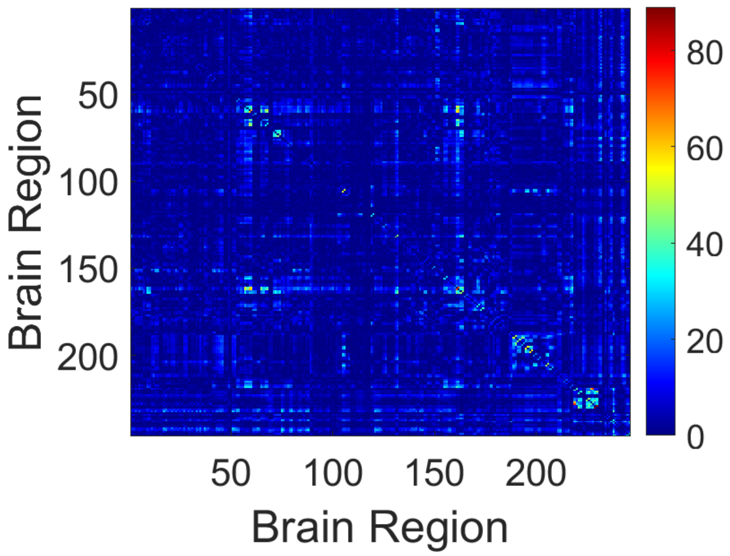

Figure 3: Original $-\log_{10}(p)$ inference matrix obtained from a two-sample test comparing the young and old groups in the UK Biobank.

Using greedy algorithm, the extracted age-related subnetwork has $|V_c| = 21$ nodes:

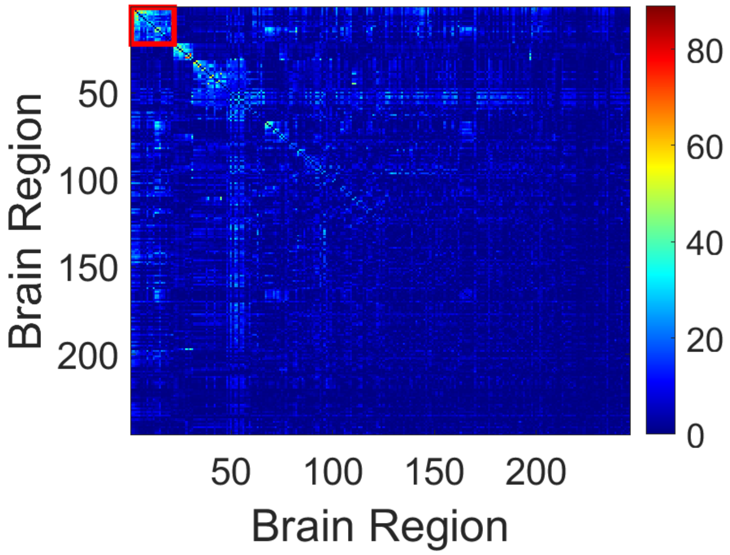

Figure 4: Identified age-related functional connectivity (FC) subnetwork, enclosed in a red rectangle, from the $-\log_{10}(p)$ inference matrix.

Based on the obtained $|V_c|$, we calculated the effect size as Cohen's $d = 0.4$, $\rho_0 = 0.03$, $\rho_1 = 0.77$, and $\sigma = 0.2$. These parameters are used as the input of the demonstration example in Section 3.1.

Results

- Classical approach (see BWAS Marek et al. (2022)): Given the derived parameters, we simulated FC matrices for varying $S_A = S_B$ subjects. According to BWAS Marek et al. (2022) and the Gitlab repository https://gitlab.com/DosenbachGreene/bwas, the power is defined as 1 - type II error after Bonferroni correction Rupert Jr et al. (2012), derived from a bootstrap subsampling procedure. As a result, with 100 bootstraps, the estimated sample size required to achieve 80% power, given the derived parameter is $S_A = S_B = 1000$.

- BNPower: By directly input the parameters to BNPower (T-test tab), we can direct calculate the required sample sizes are $S_A = S_B = 80$ to achieve at least 80% power (see Section 3.1 in the main manuscript). The corresponding code can be found in the Github repository https://github.com/bichuan0419/brain_connectome_power_tool.

5 Runtime table

Here we provided a table for the expected runtimes of BNPower on a local windows PC, for various settings of sample sizes SS and total number of brain regions N.

Runtime (sec.)	N=100	N=120	N=140	N=160	N=180	N=200	N=220	N=240
SS=100	70'	97'	152'	172'	237'	293'	392'	562'
SS=200	134'	192'	266'	351'	447'	548'	659'	773'
SS=500	419'	590'	787'	992'	1220'	1470'	1740'	2040'
SS=1000	839'	1171'	1562'	2010'	2492'	3035'	3620'	4475'

Table 1: Approximate Runtimes for various sample sizes (SS) and total number of nodes (N). Other parameters are set to be: $|V_c| = 20$, $\rho_1 = 0.7$, $\rho_0 = 0.01$, Cohen's $d = 0.6$, $\sigma = 1$, $\alpha = 0.05$, $M = 100$, and $K = 100$. The BNPower algorithm was executed on a Windows PC equipped with a 13th Gen Intel(R) Core(TM) i5-1340P processor (1.90 GHz) and 32.0 GB RAM (31.6 GB usable). The system operated on a 64-bit Windows OS with an x64-based processor. Computations were performed without the aid of a parallel computing toolbox.

6 Example networks with imported covariance and reliability matrices

To derive sample covariance matrix from the real-world dataset, the user should first obtain the stacked, vectorized functional connectivity measures for all subjects so that the complete connectivity matrix will be of size $S \times \frac{N(N-1)}{2}$, where S is the number of subjects and N is the

number of brain regions. Then the covariance matrix can be obtained by calling the covariance function (e.g., cov() in MATLAB). This will result in a covariance matrix of size $\frac{N(N-1)}{2} \times \frac{N(N-1)}{2}$. In addition, the recently developed methods for large covariance estimation with shrinkage and regularization can also be applied Fan et al. (2016a); Friedman et al. (2008); Chen et al. (2011). The test-retest reliability values that are used to assess the consistency of subjects' FC across difference sessions, are calculated using the Intraclass Correlation Coefficient (ICC) Noble et al. (2019, 2017). For UK Biobank data, we calculated the reliability values based on the those subjects who have repeated imaging measures (e.g., using the *psych* library in R).

In the following demonstration, the parameters used are $N = 100, |V_c| = 20, \rho_1 = 0.7, \rho_0 = 0.01, S_A = S_B = 50$, Cohen's $d = 0.6$, $\sigma = 1$, $\alpha = 0.05, M = 100, K = 100$.

With default settings of the covariance and reliability matrices, the example network is shown in Fig. 5.

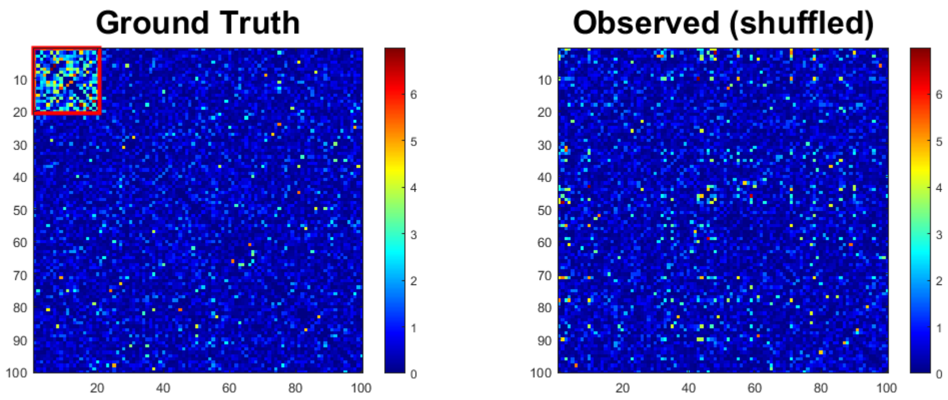

Figure 5: Example networks when the "Show Example Network" button is pushed with parameter settings: $N = 100, |V_c| = 20, \rho_1 = 0.7, \rho_0 = 0.01, S_A = S_B = 50$, Cohen's $d = 0.6$, $\sigma = 1$, $\alpha = 0.05, M = 100, K = 100$.

When users have a pre-defined covariance matrix to import (e.g., derived from existing studies), the imported covariance matrix has dimensions $NE \times NE$, where $NE = 4950$ represents the number of edges. In the example demonstrated below, we use a covariance matrix denoted as $\mathbf{C}$, whose values are derived from the UK Biobank. The example networks are shown in Fig. 6.

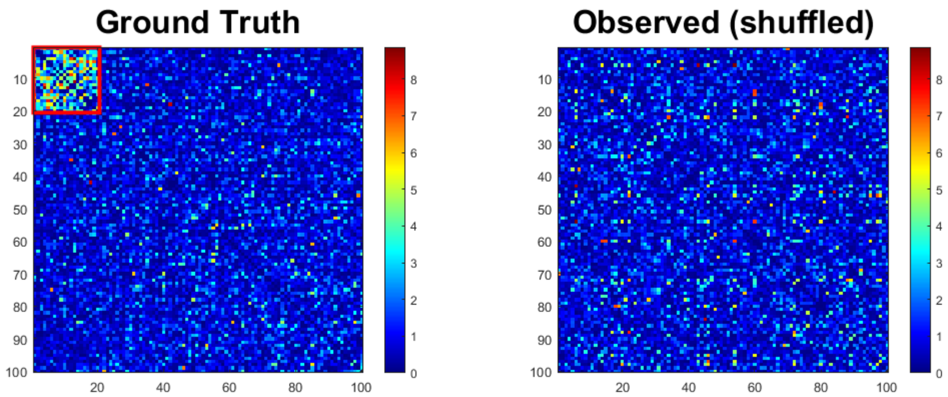

Figure 6: Example networks when the "Show Example Network" button is pushed with imported covariance matrix.

When users need to import a pre-defined reliability matrix, the imported matrix has dimensions $N \times N$. In the example demonstrated below, we used $N = 100$, resulting in the reliability matrix $\mathbf{R}$ having dimensions of 100×100. The elements R_{ij} of this matrix are derived from the UK Biobank. The example networks are shown in Fig. 7.

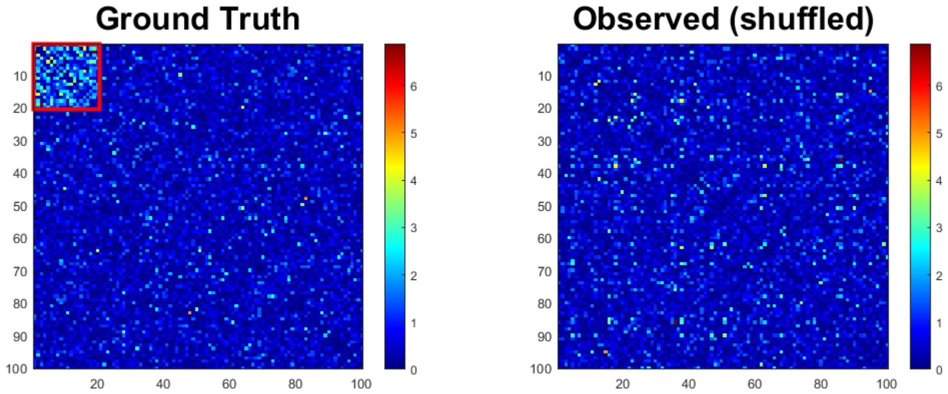

Figure 7: Example networks when the "Show Example Network" button is pushed with imported reliability matrix.

The full covariance matrix (30135×30135) and reliability matrix can be found at https://drive.google.com/drive/folders/1RPz8oaj3jpV8ocBtrvUNLkuMx3vFHDtH?usp=drive_link.

7 Power calculation comparison using various inference methods

Various methods perform statistical inference for neuroimaging data. In the data-driven network analysis of whole-brain connectomic studies, one such method is Network-based Statistics (NBS) Zalesky et al. (2010). A MATLAB script is available for users interested in comparing power calculations using BNPower and NBS. It's important to note that the main differences between NBS and BNPower are their subnetwork identification algorithms and the test statistics used for network-level inference. In BNPower, the subnetwork extraction method follows Wu et al. (2022), and its test statistic is detailed in (2.3) Chen et al. (2023). For NBS, the method identifies the largest supra-threshold connected subnetwork, with the corresponding test statistic being the size of the extracted subnetwork Zalesky et al. (2010).

To compare the calculated power using BNPower and NBS, the input parameters for generating the FC matrices are set as follows: $N = 100, |V_c| = 21, \rho_1 = 0.77, \rho_0 = 0, \sigma = 0.2, S_A = S_B = 200$, Cohen's $d = 0.4, \alpha = 0.05, M = 100, K = 100$. The sample output of the script is:

```
The power using BNPower is: 1.00, with sample sizes SA = 200, SB = 200.
The power using NBS is: 0.53, with sample sizes SA = 200, SB = 200.
```

where an example of the ground truth network is shown in Figure 8.

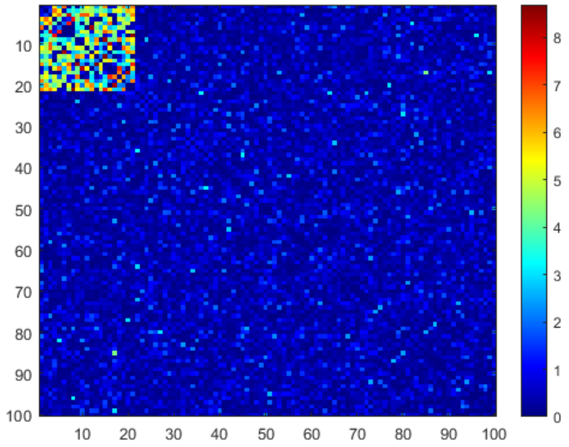

Figure 8: Example of ground truth network obtained using the input parameters: $N = 100, |V_c| = 21, \rho_1 = 0.77, \rho_0 = 0, \sigma = 0.2, S_A = S_B = 200$, Cohen's $d = 0.4, \alpha = 0.05, M = 100, K = 100$.

Users can easily modify the script to change the statistical inference methods.

8 Statistical power of detecting large, sparse predictor-of-interest related subnetwork

In the scenario that the predictor-related edges form a small subset of all the edges but cover all the node in the entire network, a non-dense and large network (approximately including all nodes) will be extracted. Consequently, the power tends to be low because the test statistic of the observed data is likely to small in the permutation test. This can be implemented by BNPower by letting the subnetwork size equal to the total number of nodes and $\rho_1 \approx \rho_0$, see Figure 9

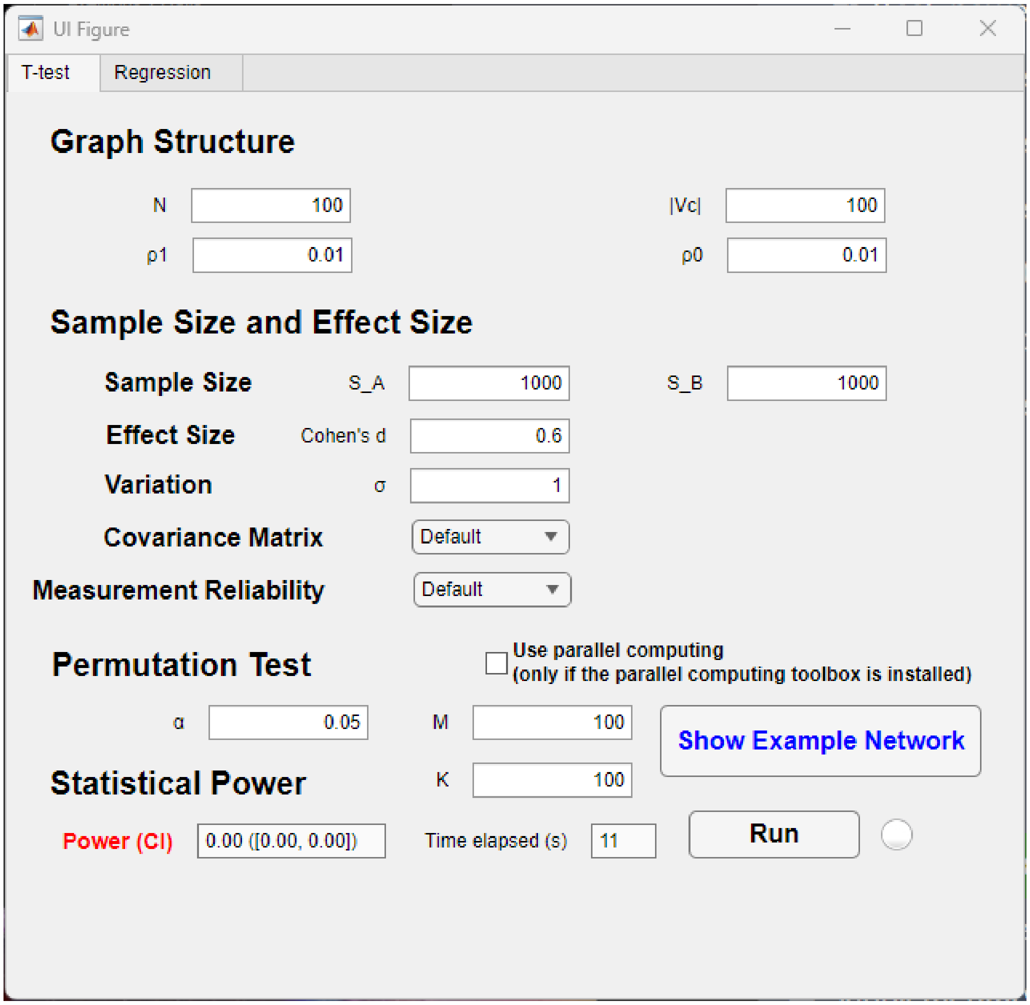

Figure 9: Example for BNPower to test the statistical power of detecting an effect in a scenario where edges associated with a predictor are sparse yet span the entire network. In the particular case, the resulting power is 0.

9 Network-level statistical power vs. edge-level inference accuracy

We also evaluate the influence of edge-level inference accuracy on power. As demonstrated by figure 10, the power increases with the accuracy.

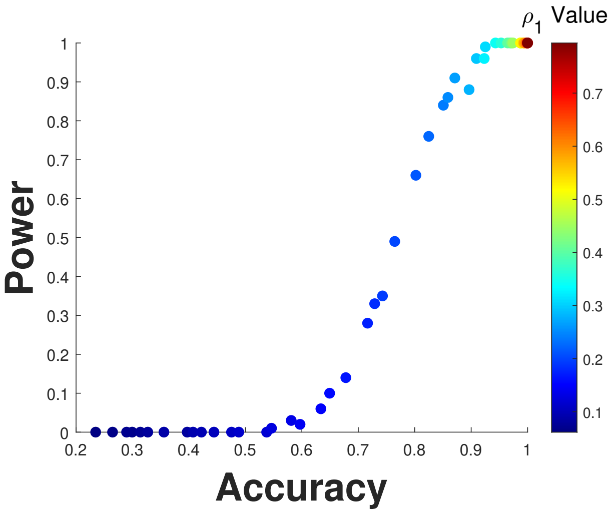

Figure 10: Network-level statistical power vs. edge-level inference accuracy as ρ_1 varies. Other parameter settings used in the two-sample test simulation study are: $N = 100, |V_c| = 20, \rho_0 = 0.01, S_A = S_B = 50, \text{Cohen's } d = 0.4, \sigma = 0.2, \alpha = 0.05, M = 100, K = 100$.

References

Alfaro-Almagro, F., Jenkinson, M., Bangerter, N. K., Andersson, J. L., Griffanti, L., Douaud, G., Sotiropoulos, S. N., Jbabdi, S., Hernandez-Fernandez, M., Vallee, E., et al. (2018). Image processing and quality control for the first 10,000 brain imaging datasets from uk biobank. *Neuroimage*, 166:400–424.

Chen, S., Zhang, Y., Wu, Q., Bi, C., Kochunov, P., and Hong, L. E. (2023). Identifying covariate-related subnetworks for whole-brain connectome analysis. *Biostatistics*, page kxad007.

Chen, Y., Wiesel, A., and Hero, A. O. (2011). Robust shrinkage estimation of high-dimensional covariance matrices. *IEEE Transactions on Signal Processing*, 59(9):4097–4107.

Cox, R. W. and Hyde, J. S. (1997). Software tools for analysis and visualization of fmri data. *NMR in Biomedicine: An International Journal Devoted to the Development and Application of Magnetic Resonance In Vivo*, 10(4-5):171–178.

Fan, J., Liao, Y., and Liu, H. (2016a). An overview of the estimation of large covariance and precision matrices. *The Econometrics Journal*, 19(1):C1–C32.

Fan, L., Li, H., Zhuo, J., Zhang, Y., Wang, J., Chen, L., Yang, Z., Chu, C., Xie, S., Laird, A. R., et al. (2016b). The human brainnetome atlas: a new brain atlas based on connectional architecture. *Cerebral cortex*, 26(8):3508–3526.

Friedman, J., Hastie, T., and Tibshirani, R. (2008). Sparse inverse covariance estimation with the graphical lasso. *Biostatistics*, 9(3):432–441.

Grabner, G., Janke, A. L., Budge, M. M., Smith, D., Pruessner, J., and Collins, D. L. (2006). Symmetric atlasing and model based segmentation: an application to the hippocampus in older adults. In *Medical Image Computing and Computer-Assisted Intervention–MICCAI 2006: 9th International Conference, Copenhagen, Denmark, October 1-6, 2006. Proceedings, Part II 9*, pages 58–66. Springer.

Harrison, D. A. and Brady, A. R. (2004). Sample size and power calculations using the noncentral t-distribution. *The Stata Journal*, 4(2):142–153.

Jenkinson, M., Bannister, P., Brady, M., and Smith, S. (2002). Improved optimization for the robust and accurate linear registration and motion correction of brain images. *Neuroimage*, 17(2):825–841.

Jenkinson, M., Beckmann, C. F., Behrens, T. E., Woolrich, M. W., and Smith, S. M. (2012). Fsl. *Neuroimage*, 62(2):782–790.

Marek, S., Tervo-Clemmens, B., Calabro, F. J., Montez, D. F., Kay, B. P., Hatoum, A. S., Donohue, M. R., Foran, W., Miller, R. L., Hendrickson, T. J., et al. (2022). Reproducible brain-wide association studies require thousands of individuals. *Nature*, 603(7902):654–660.

Miller, K. L., Alfaro-Almagro, F., Bangerter, N. K., Thomas, D. L., Yacoub, E., Xu, J., Bartsch, A. J., Jbabdi, S., Sotiropoulos, S. N., Andersson, J. L., et al. (2016). Multimodal population brain imaging in the uk biobank prospective epidemiological study. *Nature neuroscience*, 19(11):1523–1536.

Mo, C., Ye, Z., Ke, H., Lu, T., Canida, T., Liu, S., Wu, Q., Zhao, Z., Ma, Y., Hong, L. E., et al. (2021). A new mendelian randomization method to estimate causal effects of multivariable

brain imaging exposures. In *PACIFIC SYMPOSIUM ON BIOCOMPUTING 2022*, pages 73–84. World Scientific.

Noble, S., Scheinost, D., and Constable, R. T. (2019). A decade of test-retest reliability of functional connectivity: A systematic review and meta-analysis. *Neuroimage*, 203:116157.

Noble, S., Spann, M. N., Tokoglu, F., Shen, X., Constable, R. T., and Scheinost, D. (2017). Influences on the test–retest reliability of functional connectivity mri and its relationship with behavioral utility. *Cerebral cortex*, 27(11):5415–5429.

Rupert Jr, G. et al. (2012). Simultaneous statistical inference.

Salimi-Khorshidi, G., Douaud, G., Beckmann, C. F., Glasser, M. F., Griffanti, L., and Smith, S. M. (2014). Automatic denoising of functional mri data: combining independent component analysis and hierarchical fusion of classifiers. *Neuroimage*, 90:449–468.

Sudlow, C., Gallacher, J., Allen, N., Beral, V., Burton, P., Danesh, J., Downey, P., Elliott, P., Green, J., Landray, M., et al. (2015). Uk biobank: an open access resource for identifying the causes of a wide range of complex diseases of middle and old age. *PLoS medicine*, 12(3):e1001779.

Thomas Yeo, B., Krienen, F. M., Sepulcre, J., Sabuncu, M. R., Lashkari, D., Hollinshead, M., Roffman, J. L., Smoller, J. W., Zöllei, L., Polimeni, J. R., et al. (2011). The organization of the human cerebral cortex estimated by intrinsic functional connectivity. *Journal of neurophysiology*, 106(3):1125–1165.

Woo, C.-W., Krishnan, A., and Wager, T. D. (2014). Cluster-extent based thresholding in fmri analyses: pitfalls and recommendations. *Neuroimage*, 91:412–419.

Wu, Q., Huang, X., Culbreth, A. J., Waltz, J. A., Hong, L. E., and Chen, S. (2022). Extracting brain disease-related connectome subgraphs by adaptive dense subgraph discovery. *Biometrics*, 78(4):1566–1578.

Zalesky, A., Fornito, A., and Bullmore, E. T. (2010). Network-based statistic: identifying differences in brain networks. *Neuroimage*, 53(4):1197–1207.
